# Ecological selectivity and the evolution of mammalian substrate preference across the K–Pg boundary

**DOI:** 10.1002/ece3.8114

**Published:** 2021-10-11

**Authors:** Jonathan J. Hughes, Jacob S. Berv, Stephen G. B. Chester, Eric J. Sargis, Daniel J. Field

**Affiliations:** ^1^ Department of Ecology & Evolutionary Biology Cornell University Ithaca New York USA; ^2^ Department of Ecology & Evolutionary Biology University of Michigan Ann Arbor Michigan USA; ^3^ University of Michigan Museum of Paleontology University of Michigan Ann Arbor Michigan USA; ^4^ Department of Anthropology Brooklyn College City University of New York Brooklyn New York USA; ^5^ Department of Anthropology The Graduate Center City University of New York New York New York USA; ^6^ New York Consortium in Evolutionary Primatology New York New York USA; ^7^ Department of Anthropology Yale University New Haven Connecticut USA; ^8^ Divisions of Vertebrate Paleontology and Vertebrate Zoology Yale Peabody Museum of Natural History New Haven Connecticut USA; ^9^ Yale Institute for Biospheric Studies New Haven Connecticut USA; ^10^ Department of Earth Sciences University of Cambridge Cambridge UK; ^11^ Museum of Zoology University of Cambridge Cambridge UK

**Keywords:** ancestral state reconstruction, euarchontans, marsupials, paleoecology, placentals, substrate use

## Abstract

The Cretaceous–Paleogene (K–Pg) mass extinction 66 million years ago was characterized by a worldwide ecological catastrophe and rapid species turnover. Large‐scale devastation of forested environments resulting from the Chicxulub asteroid impact likely influenced the evolutionary trajectories of multiple clades in terrestrial environments, and it has been hypothesized to have biased survivorship in favour of nonarboreal lineages across the K–Pg boundary. Here, we evaluate patterns of substrate preferences across the K–Pg boundary among crown group mammals, a group that underwent rapid diversification following the mass extinction. Using Bayesian, likelihood, and parsimony reconstructions, we identify patterns of mammalian ecological selectivity that are broadly similar to those previously hypothesized for birds. Models based on extant taxa indicate predominant K–Pg survivorship among semi‐ or nonarboreal taxa, followed by numerous independent transitions to arboreality in the early Cenozoic. However, contrary to the predominant signal, some or all members of total‐clade Euarchonta (Primates + Dermoptera + Scandentia) appear to have maintained arboreal habits across the K–Pg boundary, suggesting ecological flexibility during an interval of global habitat instability. We further observe a pronounced shift in character state transitions away from plesiomorphic arboreality associated with the K–Pg transition. Our findings are consistent with the hypothesis that predominantly nonarboreal taxa preferentially survived the end‐Cretaceous mass extinction, and emphasize the pivotal influence of the K‐Pg transition in shaping the early evolutionary trajectories of extant terrestrial vertebrates.

## Graphical abstract

We explore the evolution of mammal ecology across the end‐Cretaceous mass extinction event, 66 million years ago. Our results suggest that nonarboreal mammals were selectively favored across this mass extinction event, congruent with the hypothesis that forest communities were devastated in the aftermath of the asteroid impact at the end of the Cretaceous.

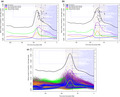

## 1 INTRODUCTION

The Cenozoic Era is colloquially known as the “Age of Mammals,” and the modern world is populated by over 6,000 extant mammalian species exhibiting an extraordinary diversity of forms and ecologies (Burgin et al., 2018; Nowak, 1999). Numerous authors have noted that the evolutionary history of extant mammalian biodiversity may have been shaped by the Cretaceous–Paleogene (K–Pg) transition, an interval that is associated with a complex set of mammalian extinctions, radiations, and shifts in species richness (Archibald, 2011; Benevento et al., 2019; Brocklehurst et al., 2021; Clemens, 2002; Wilson et al., 2014). However, the precise influence of the K–Pg transition on the rate, timing, and nature of mammalian diversification is contentious and may have varied among major mammalian lineages (Bininda‐Emonds et al., 2007; Chen et al., 2019; Grossnickle et al., 2019; Halliday et al., 2016; Hedges et al., 1996; O'Leary et al., 2013; Phillips, 2016; Pires et al., 2018; Springer et al., 2003; Wible et al., 2007).

Even in the best‐sampled North American localities, a comprehensive, direct assessment of global patterns of mammalian ecological changes across the K–Pg boundary is precluded by the relatively sparse mammalian fossil record in the latest Cretaceous and earliest Paleogene (Davies et al., 2017), though strong patterns of ecological selectivity are expected in light of high estimated rates of mammalian extinction (Grossnickle & Newham, 2016; Longrich et al., 2016; Wilson, 2013). Surviving mammalian lineages appear to have undergone rapid morphological diversification from primarily small insectivorous or omnivorous forms, and they colonized a wide range of vacant ecological niches in the aftermath of the mass extinction event (Alroy, 1999; Grossnickle et al., 2019; Halliday & Goswami, 2016a; Lyson et al., 2019; O'Leary et al., 2013; Shelley et al., 2021; Smith et al., 2010; Wilson, 2014). Theoretical studies have predicted that fossorial and semi‐aquatic mammals may have had a selective advantage across the K–Pg boundary because their substrate preferences would have shielded them from the severe, short‐term effects of the Chicxulub asteroid impact such as a hypothesized heat pulse and associated wildfires (DeBey & Wilson, 2017; Robertson et al., 2004). Alongside global fires and longer‐term climatic effects, the asteroid impact resulted in forest devastation on a global scale (Carvalho et al., 2021; Field et al., 2018; Lyson et al., 2019; Nichols & Johnson, 2008; Tschudy et al., 1984; Vajda et al., 2001) and substantially altered floral communities for centuries (Carvalho et al., 2021; Wilf & Johnson, 2004). Recent work on birds suggested that the collapse of global forests drove arboreal Mesozoic avialans to extinction at the K–Pg boundary, with multiple subsequent originations of arboreal habits arising among crown birds once forests had recovered (Field et al., 2018).

Here, we investigate patterns of substrate preference evolution across crown group mammals—another major K–Pg boundary‐crossing terrestrial vertebrate clade. First, we assessed the evidence for whether mammals were subject to comparable habitat‐related selectivity across the K–Pg boundary. We performed ancestral state reconstructions (ASRs) of substrate preferences on alternative phylogenetic hypotheses for extant mammals (Meredith et al., 2011; Upham et al., 2019). Though not definitive, when interpreted within the context of available fossil evidence, we consider the results suggestive of a pattern of predominant K–Pg survivorship among semi‐arboreal or nonarboreal mammals, with extant mammalian clades characterized by obligately arboreal ecologies generally arising in the early Cenozoic. Second, we examined the relative clade‐wide frequencies of particular evolutionary transitions throughout the evolutionary history of Mammalia using a model‐based approach. Our analyses identify an interval early in placental mammal evolutionary history marked by a striking increase in inferred transitions toward nonarboreality. Notably, this interval of apparent clade‐wide directional selectivity toward nonarboreality aligns with plausibly K–Pg‐associated cladogenesis among crown placentals, although we note that the divergence times of early placental clades remain contentious. Acknowledging these lingering divergence time uncertainties, we contend that our analyses help illuminate the hidden influence of the K–Pg transition on major ecological patterns early in the evolutionary history of placental mammals.

## 2 MATERIAL AND METHODS

### 2.1 Character state assignment

All 164 mammalian lineages from the time‐scaled phylogenetic hypothesis of Meredith et al. (2011), representing most extant family‐level phylogenetic diversity, were assigned an ecological character state of arboreal, semi‐arboreal, or nonarboreal (electronic supplementary material). Character states reflect where mammals form nests or otherwise reside. More explicitly, we characterize a “nest” as a construct used for: rearing young, resting, or sleeping (examples include the leaf nests of gorillas or the dreys of squirrels). Alternatively, a mammal may reside in a tree without construction of a nest, where its “residence” is primarily used for sleeping or resting, and may involve rearing young but does not involve any structural modifications to the tree (sloths, for example, often find a leafy area in a tree to sleep in but do not modify the tree or its foliage). An arboreal mammal is therefore one that, in the wild, will virtually always reside or nest in a living tree, be it among the branches or in an existing tree cavity. To be classed as semi‐arboreal, the mammal in question will often reside or nest in a living tree in the wild but does not do so exclusively. In general, for a semi‐arboreal mammal, trees are convenient but not essential, and another substrate (e.g., a rock face) may provide a suitable alternative. All species that fall outside these definitions are classed as nonarboreal, such that the mammal in question does not nest or reside in trees at all, or only does so incidentally in a small number of documented cases. We believe this coding strategy is conservative with respect to mammals that exhibit an obligately arboreal ecology for nesting and residence, and it allows us to discriminate among lineages with obligately arboreal habits from those that occupy trees facultatively or opportunistically.

### 2.2 Alternative phylogenetic frameworks

In order to assess the influence of phylogenetic uncertainty on our ancestral ecological reconstructions, we evaluated them with respect to well‐supported phylogenetic hypotheses from Meredith et al. (2011) as well as the node‐dated maximum clade credibility consensus tree from Upham et al. (2019) and its associated posterior distribution of tree topologies. Both phylogenetic hypotheses are derived from a supermatrix inference approach, with Upham et al. (2019) using sequences for 31 genes (building on the 26 from Meredith et al., 2011). Meredith et al. (2011) used a family‐level approach to build a time‐calibrated tree of 164 mammalian lineages, of which 142 were single species, 16 were congeneric chimaerics, and six were chimaerics above the genus level. Upham et al. (2019) employed a method that separated phylogenetic inference into divergences between major lineages (“backbone”) and clades at the species level (“patch”) (Jetz et al., 2012; Mishler, 1994) to generate a phylogeny uniting ~4,100 species. Our analysis scores the subset of taxa in the Upham et al. (2019) dataset that matched the taxon set from the Meredith et al. (2011) analysis. This yielded two complementary phylogenetic consensus topologies with the same taxon set, on which we estimated character evolution. In the 12 cases where the Upham et al. (2019) dataset did not contain the same species as in Meredith et al. (2011), we replaced the missing species with its closest relative with the same character state (Table S1). By considering these alternative hypotheses, we specifically assess how robust our inferences are to areas of conflict between the two consensus topologies, such as the monophyly of Euarchonta (Primates + Scandentia + Dermoptera; Upham et al., 2019) and the placement of Scandentia as the sister group to Glires (Rodentia + Lagomorpha; Meredith et al., 2011). Upham et al. (2019) cite posterior probabilities of 0.96 for the monophyly of Euarchonta and 0.78 for Dermoptera + Scandentia. Meredith et al. (2011) found that DNA and amino acid trees agree on the monophyly of Scandentia + Glires but with bootstrap support of <90%.

### 2.3 Model selection

We assessed the relative fit of three alternative time‐homogeneous transition models with maximum likelihood in the ape (Paradis et al., 2004) and phytools (Revell, 2012) *R* packages (R Core Team, 2014) on each consensus tree. Following Field et al. (2018), one model comprised two rates, such that transitions among all three character states (arboreal, semi‐arboreal, and nonarboreal) were permitted, but transitions to and from semi‐arboreality were allowed a different rate from transitions that bypass this intermediate stage. A second model comprised four rates such that transitions from nonarboreal to arboreal were required to pass through semi‐arboreality, with separate forward and reverse rates for each pair of state transitions. These models reflect the presumed biological reality that transitioning from nonarboreality to arboreality or vice versa through an intermediate state likely occurs at a different rate than transitions lacking an intermediate state. We also tested a third maximally parameterized (six‐rate) model (“ARD”—all rates different) in which forward and reverse rates were allowed to vary across all states.

Hidden Markov models (HMMs) have emerged as a powerful tool for assessing the possibility that unobserved rate heterogeneity can have an outsized influence on reconstructing the evolutionary history of discrete characters (Beaulieu & O'Meara, 2016; Beaulieu et al., 2013; Boyko & Beaulieu, 2021). In comparison with time‐homogeneous models, which assume that specified character transition rates do not evolve, HMMs provide an elegant solution for evaluating the hypothesis that the mode of character evolution has evolved throughout a clade's evolutionary history. To assess this possibility in our data, we generated three HMMs using the corHMM *R* package (Beaulieu et al., 2013). Our initial analysis of time‐homogeneous models revealed that the six‐rate ARD and four‐rate intermediate model were preferred (Table 1). Therefore, we elected to compare three HMMs based on those models. The first of these consisted of a model that included two‐rate classes: one with an ARD model and one with the four‐rate model. The second and third HMMs reflected ARD models with two or three rate classes, respectively. In all cases, we assumed symmetric transition rates among rate classes. As time‐homogeneous models are a special case of HMMs (reflecting one rate class), we compared all evaluated models with the Akaike information criterion (AIC).

**TABLE 1:** Akaike information criterion (AIC) scores for all models evaluated on both the Meredith et al. (2011) and Upham et al. (2019) consensus topologies, indicating that the four‐rate model is preferred (lowest AIC score, highlighted gray)

Model	Meredith et al. (2011)	Upham et al. (2019)
2 rate	244.93	244.22
4 rate	231.92	234.30
6 rate	235.90	238.67
HRM 4 rate, 2 cat	245.64	246.21
HRM 6 rate, 2 cat	249.64	250.56
HRM 6 rate, 3 cat	268.69	270.37

### 2.4 Reconstructing the evolution of mammalian arboreality

We performed likelihood‐based ancestral state reconstructions (ASRs) in *R* (R Core Team, 2014). We used the ace() likelihood function in ape (Paradis et al., 2004) and a customized implementation of Bayesian stochastic mapping, described below (Bollback, 2006; Revell, 2012). We also performed maximum parsimony reconstructions using the ancestral.pars() function in the *R* package phangorn (Schliep, 2011).

As part of the VertLife initiative (http://vertlife.org/data/mammals/), Upham et al. (2019) provided a set of 10,000 credible phylogenetic trees sampled from the Bayesian posterior distribution estimated in that study. Therefore, for analyses based on the Upham et al. (2019) consensus tree, we leveraged this resource to account for stochastic uncertainty in branch lengths and tree topology. For each of the time‐homogeneous models we evaluated, we performed a Bayesian stochastic character mapping analysis across 1,000 sampled trees from the Upham et al. (2019) posterior distribution, and we estimated 500 stochastic character maps on each. These results were then summarized with respect to the Upham et al. (2019) consensus tree. For analyses directly using the consensus trees, we estimated 5,000 stochastic maps.

To make this task computationally tractable, we generated new *R* code to perform these analyses in parallel across multiple CPUs using the “parallel” (R Core Team, 2014), “doSNOW” (Wallig et al., 2020a), and “doParallel” (Wallig et al., 2020b) *R* libraries. Our approach (see simmap_parallel.R; https://github.com/jakeberv/mammal_arboreality) operates on “phylo” or “multiPhylo” tree objects, accelerating several aspects of this analysis. The wrapper function simmap.parallel() takes minimally as arguments a tree or set of trees, a discrete character dataset, a time‐homogeneous model, and a specified assumption about the distribution of character states at the root (optionally equal or following the FitzJohn et al. (2009) root state prior). Briefly, the function first estimates a Q matrix for each of the trees that are passed to it, using fitMK() (Revell, 2012), or alternatively accepts an external Q matrix estimate. Then, depending on the options selected, simmap.parallel() generates stochastic character maps on each of the provided trees using fastSimmap() from the *R* package ratematrix (Caetano & Harmon, 2017), the estimated Q matrix for each tree, and the stated root prior. Lastly, a final combined multiSimmap object is generated. This output can be parsed by phytools::describe.simmap() with the argument ref.tree set to the target consensus tree on which to summarize the results. We provide additional code to accelerate aspects of this summation in a modified function describe.simmap.alt(), which can otherwise be very time‐consuming for large numbers of trees (Eliot Miller, personal communication, March 2021).

### 2.5 Investigating clade‐wide temporal patterns in character transition rates

In addition to individual node and branch reconstructions, we examined the relative frequencies of particular transition types through time across the two consensus trees as well as the posterior tree distribution from Upham et al. (2019). For example, in a two‐rate bidirectional model with two states, forward and reverse transition rates can be time‐homogeneous, while the total counts of particular transition events across all branches vary through time and depend on the structure of the underlying phylogeny. Revell (2017) outlined an approach for visualizing the history of clade‐wide changes in character transitions for a discrete character model under stochastic mapping. This approach first takes a stochastic character mapping simulation and partitions the underlying tree into a specified number of time bins. The average number of character transitions across branches and simulations is calculated within each time bin, and this value is then normalized for patterns of cladogenesis by dividing by the total branch length within a time bin. Revell's (2017) example provides a pragmatic solution for visualizing the behavior of a discrete character model through time in the context of stochastic character mapping.

Here, we refine this approach to allow examination of temporal patterns in the relative frequencies of each transition type from a given model (see rate_through_time.R; https://github.com/jakeberv/mammal_arboreality). We generate visualizations for stochastic character mapping under the optimal models for the Meredith et al. (2011) and Upham et al. (2019) consensus topologies, as well as for a sample of 1,000 posterior trees from Upham et al. (2019). These visualizations allow us to further examine the hypothesis that patterns of clade‐wide trends in transitions toward and away from arboreality may have been influenced by the K–Pg transition.

## 3 RESULTS

### 3.1 Node reconstructions

Under the preferred four‐rate model (Table 1), stochastic mapping supports a pattern whereby arboreality emerged repeatedly and independently among several different clades following the K–Pg mass extinction. We detect at least 10 instances of post‐K–Pg transitions to arboreality under the Meredith et al. (2011) framework (Figure 1) and 11 cases across the Upham et al. (2019) dataset (Figure 2). These general patterns hold across both alternative topologies and under parsimony and likelihood optimality criteria (Figures S1‐S18).

**FIGURE 1:**
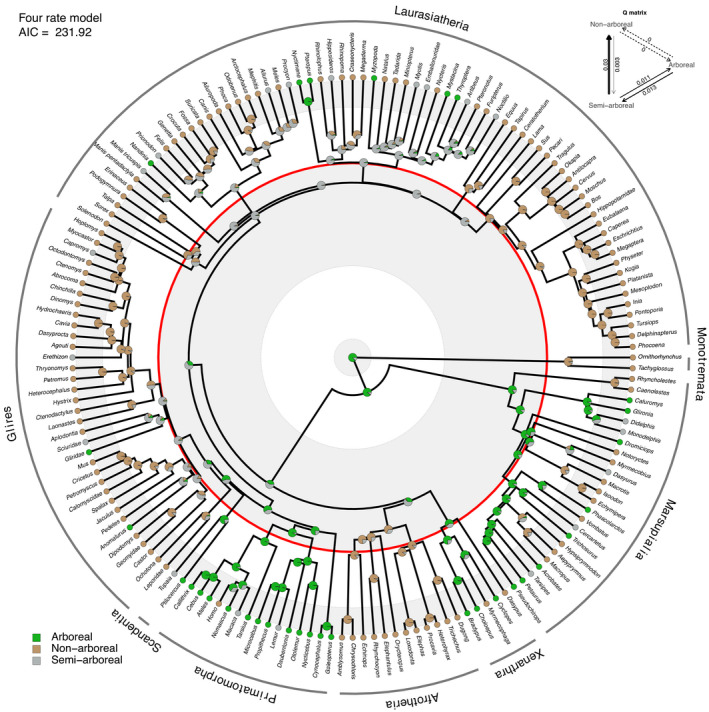
Bayesian ancestral state reconstruction of substrate preference in mammals using the Meredith et al. (2011) consensus tree. Pie charts indicate posterior probabilities of character states from SIMMAP under the four‐rate model. The red circle indicates the K–Pg boundary. Primatomorpha and Marsupialia are recovered as having retained predominantly arboreal habits across the boundary, and all other mammalian clades are reconstructed with a majority probability of semi‐ or non‐arboreality across the K–Pg boundary. Inset top right is a depiction of the maximum likelihood estimate of the Q matrix, indicating non‐zero values for each of the four allowed rates

**FIGURE 2:**
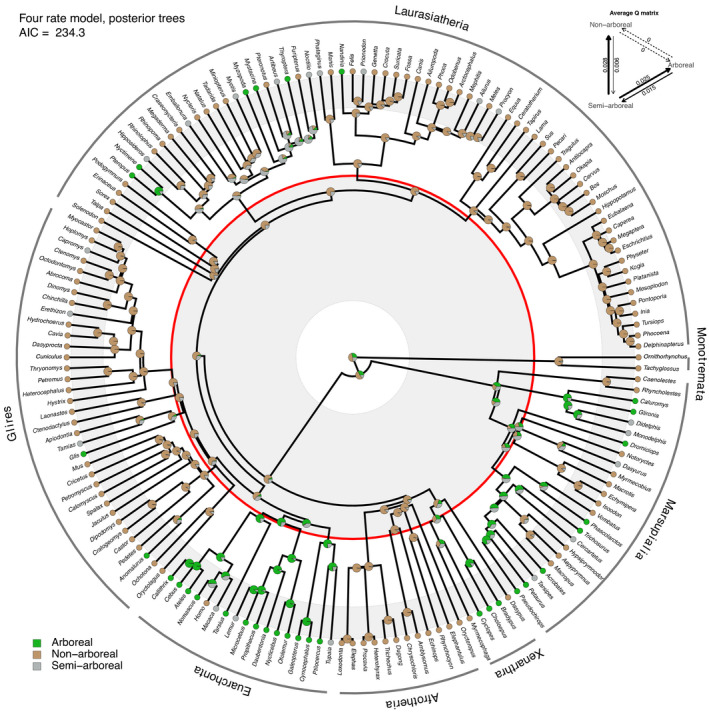
Bayesian ancestral state reconstruction of substrate preference in mammals under the four‐rate model, displayed on the Upham et al. (2019) consensus tree. Pie charts indicate summaries of analyses performed on 1,000 sampled trees from the Upham et al. (2019) posterior distribution of tree topologies. The character state probabilities for each node are assessed relative to all phylogenetically equivalent nodes in each tree from the Upham et al. (2019) posterior sample. The red circle indicates the K–Pg boundary. Euarchonta is recovered as arboreal across the K‐Pg boundary, whereas Marsupialia is recovered as semi‐arboreal and all other clades are primarily non‐arboreal across the boundary. Inset top right is a depiction of the average maximum likelihood estimate of the Q matrix, indicating non‐zero values for each of the four allowed rates

Bayesian stochastic mapping under the flexible ARD model suggests that state transitions that pass through a semi‐arboreal intermediate are detected more frequently than direct transitions from arboreality to nonarboreality or vice versa (Figures S6, S9, and S12). Additionally, the ARD model detects no direct transitions from nonarboreality to arboreality. By contrast, in the two‐rate model, direct transitions from nonarboreality to arboreality are detected at a higher frequency than the reverse, while transitions away from semi‐arboreality occur at an intermediate frequency (Figures S2, S8, and S11). We interpret these results to suggest that the transitions inferred under the ARD model are more biologically plausible than those under the two‐rate model.

Under both the Meredith et al. (2011) and the Upham et al. (2019) consensus topologies, the preferred four‐rate and ARD models reconstructed more nodes near the K–Pg boundary as semi‐arboreal than did the two‐rate model, especially on the Meredith et al. topology (Figures S5, S6, S15, and S16). Incorporating a sample of 1,000 tree topologies from the posterior distribution of Upham et al. (2019) made little difference in stochastic mapping reconstructions under the two‐rate or ARD models (Figures S15‐S18). However, for the optimal four‐rate model, consideration of posterior topological uncertainty leads to a marked increase in circum K–Pg nodes being recovered as nonarboreal rather than semi‐arboreal (compare Figure 2 to Figure S14). We suggest this is a consequence of more pronounced changes in the average estimated Q matrix (inset in Figure 2) observed for the four‐rate model when compared to the two‐rate or ARD models, summarized across the posterior tree sample. Although both sets of reconstructions are generally consistent with the hypothesis of K–Pg‐associated selectivity against arboreality, it is clear that considering information from the Upham et al. (2019) posterior tree set impacts the interpretation of our node state reconstructions.

The overall signal we detect is consistent with the hypothesis of predominant survivorship of nonarboreal or semi‐arboreal mammals across the K–Pg boundary: few lineages reconstructed as predominantly arboreal are inferred to have survived the K–Pg mass extinction. However, our analyses also highlight two possible exceptions: euarchontans and marsupials. On the Meredith et al. (2011) topology under all models, early members of total‐clade Primatomorpha (Primates + Dermoptera) are inferred to have either retained arboreal habits across the K–Pg boundary (Figure 1; Figures S4‐S6) or acquired arboreality shortly thereafter (see below). On the Upham et al. (2019) consensus topology, in which Euarchonta (Primates + Dermoptera + Scandentia) is inferred to be monophyletic, arboreality is reconstructed as having arisen along the euarchontan stem lineage in all models (Figures S13‐S16). Considering posterior topological uncertainty also leads to Euarchonta being reconstructed as arboreal at the time of the K–Pg transition, whereas the majority of other lineages are reconstructed as nonarboreal under the four‐rate model and semi‐arboreal otherwise (Figure 2; Figures S17 and S18). Although not supported by Meredith et al. (2011), a monophyletic Euarchonta has frequently been supported by other phylogenetic analyses (Chester et al., 2015, 2017; O'Leary et al., 2013; Springer, 2004; Springer et al., 2003). Under parsimony and two likelihood models (four‐rate and ARD), most marsupials are additionally reconstructed as having retained arboreal habits across the K–Pg boundary, or acquired them shortly thereafter (Figure 1; Figures S4‐S6, S13‐S16). However, this signal is diminished when considering the Upham et al. (2019) distribution of topologies (Figure 2).

### 3.2 Clade‐wide temporal patterns in character transition rates

**FIGURE 3:**
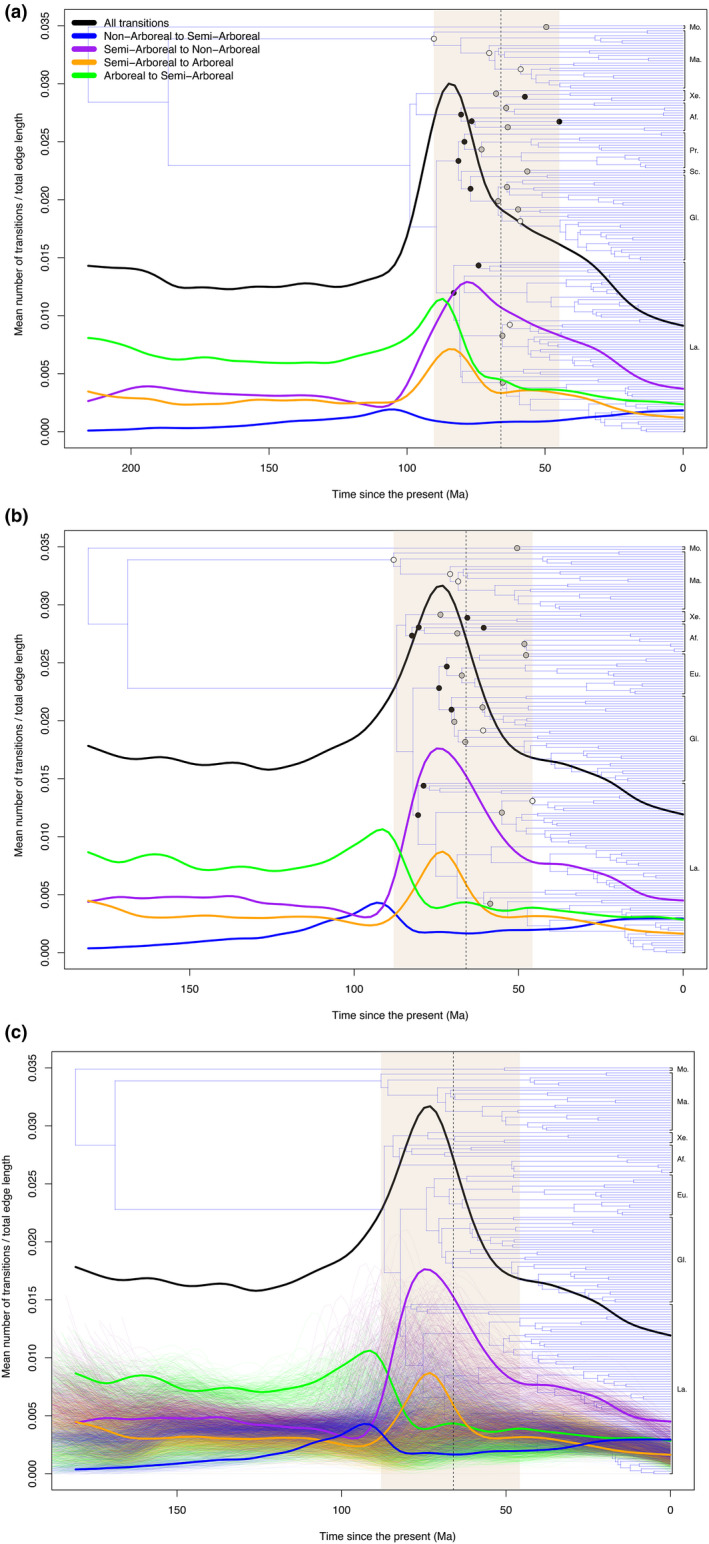
Transition “rate‐through‐time” plots for the A) Meredith et al. (2011) and B) Upham et al. (2019) consensus topologies as derived from the four‐rate model. The K‐Pg boundary is indicated with a vertical dashed line. Highlighted nodes indicate ordinal or higher divergences that are plausibly associated with the K‐Pg boundary; as summarized in Supplemental Table 3 from Upham et al. (2019): K–Pg‐associated nodes from Meredith et al. (2011) are marked in white, from Upham et al. (2019) in black, and if from both, grey. The beige shaded area indicates the time span of these K‐Pg associated nodes, encompassing many more nodes than are highlighted. Each colored curve represents the relative frequency of state transitions of a given type within each of 50 equal‐sized time bins across 5,000 simulations. The black curve indicates a summary of all transition types. A clear spike in state transitions is visibly associated with circum‐K‐Pg nodes, with semi‐arboreal to non‐arboreal transitions representing the dominant transition type across the clade. C) The results of 500 stochastic maps simulated on each of 1,000 trees sampled from the posterior distribution of Upham et al. (2019). One curve is drawn for each posterior tree, color coded to match those shown in panel A and B. In A and B; Mo=Monotremata, Ma=Marsupialia, Xe=Xenarthra, Af=Afrotheria, Pr=Primatomorpha, Sc=Scandentia, Gl=Glires, La=Laurasiatheria, Eu=Euarchonta

For both the Meredith et al. (2011) and Upham et al. (2019) consensus topologies, the highest frequency of character transitions detected by the optimal four‐rate model falls within the range of divergence time uncertainty for many clades whose originations have been proposed to be associated with the K–Pg boundary (see Discussion). Moreover, the temporal sequence of peaks in the relative frequencies of particular character transition types appears to be consistent with the hypothesis of selection against obligate arboreality leading up to and through the K–Pg boundary (i.e., transitions away from arboreality, followed by transitions toward arboreality, at least as indicated by analyses on the Upham et al. (2019) consensus topology). These patterns are similar for analyses performed on the Meredith et al. (2011) (Figure 3a) and Upham et al. (2019) (Figure 3b) consensus topologies, as well as the Upham et al. (2019) posterior tree sample (Figure 3c). Tracking fluctuations in the relative frequencies of mammalian ecological transitions approaching the K–Pg boundary (Figure 3), the four‐rate model first detects a slight uptick and subsequent reduction in clade‐wide transitions from arboreal to semi‐arboreal character states, which remains low to the present. This initial pulse is followed by (or is perhaps concurrent with) a large peak in transitions from semi‐arboreal to nonarboreal character states, which declines gradually to the present. This peak of character transitions toward nonarboreality appears stronger in the analyses employing the Upham et al. (2019) topologies than in the analyses using the Meredith et al. (2011) consensus topology. Subsequently, a peak in clade‐wide transitions from semi‐arboreal to arboreal character states is detectable in both analyses, which returns to pre‐K–Pg levels. Temporal patterns of character state changes from nonarboreal to semi‐arboreal appear relatively flat in the Meredith et al. (2011) topology, with a stronger uptick associated with other peaks in the Upham et al. (2019) topologies. When interpreting these results, it should be noted that only one type of character transition can occur at a given time on a given branch on a given stochastic map. Therefore, an apparent increase in one type of character state transition may necessarily be associated with a decline in the frequency of a different type of character state transition.

These patterns emphasize that the most dramatic clade‐wide mode changes appear to be associated with the interval encompassing many clade originations hypothesized to be related to the K–Pg transition. These results suggest that the early diversification of placental mammals was associated with clade‐wide shifts in the relative rates of character transitions toward and away from particular ecological strategies and that the sequence of these shifts is consistent with the hypothesis that the transient loss of available arboreal habitats at the K–Pg boundary may have driven those changes. Although the presently wide uncertainty in divergence times precludes a definitive statement, it is important to note that if our documented peaks in evolutionary transitions did occur during the Cretaceous, they could be consistent with the “Early Rise Hypothesis.” In that scenario, an ecological radiation of mammals began prior to the Cretaceous–Paleogene transition, potentially associated with concomitant diversification events among angiosperms and selected groups of insects (Grossnickle et al., 2019).

## 4 DISCUSSION

### 4.1 Inference from the fossil record

Our ancestral state reconstructions consistently support survivorship patterns favoring predominantly nonarboreal or semi‐arboreal substrate use across the K–Pg boundary, under likelihood, Bayesian, and parsimony models. This is consistent with previous ASR approaches that recover early mammalian nodes as mostly nonarboreal until just after the K‐Pg boundary (Wu et al., 2017). With few exceptions (Lyson et al., 2019), well‐preserved mammalian fossils from close to the K–Pg boundary and the first ca. one million years of the extinction's aftermath are exceedingly rare (Hartman, 2002; Lofgren et al., 2004; Williamson, 1996; Wilson et al., 2014). Most fossils known from this interval are too fragmentary to robustly inform reconstructions of substrate preferences. Indeed, even in cases where strong inferences about the predominant substrate use of a fossil taxon can be drawn, uncertainty regarding parameters such as nesting behaviors is unavoidable. Uncertainty surrounding the phylogenetic position of such fossils presents further challenges with respect to interpreting their implications for early ecological transitions among crown placentals (Halliday et al., 2017). Accepting these limitations, our reconstructions are consistent with the preferential survivorship of nonarboreal mammals across the K–Pg mass extinction. In contrast to evolutionary patterns among crown birds, in which strong selection for nonarboreal ecologies appears to be unambiguously supported by both phylogenetic and fossil evidence (Field et al., 2018, 2020), definitive assessments of selective patterns among K–Pg boundary‐crossing mammals will remain elusive in the absence of additional fossil evidence. Until that time, we interpret our results in the context of the currently known circum K–Pg mammalian fossil record, as well as the more complete records from earlier and later in mammalian evolutionary history.

Based on postcranial morphology, some early (ca. 125 Ma) therians including *Eomaia* (Ji et al., 2002), *Ambolestes* (Bi et al., 2018), and *Sinodelphys* (Luo et al., 2003), have been interpreted as arboreal or scansorial, as has the oldest known therian, the ca. 160 Ma *Juramaia* (Luo et al., 2011). Mammalian arboreality may have been common in the Mesozoic, concurrent with increasing mammalian locomotor diversity (Chen & Wilson, 2015; Grossnickle et al., 2019). In contrast, later pre‐K–Pg lineages for which locomotor reconstructions are possible, such as the metatherian *Asiatherium* (Trofimov & Szalay, 1994) and the eutherians *Barunlestes* and *Zalambdalestes* (Chester et al., 2010, 2012; Kielan‐Jaworowska, 1978) are not interpreted to have been arboreal (Chen & Wilson, 2015). Inclusion of Mesozoic fossil taxa in our reconstructions would likely inflate posterior estimates for early arboreality among mammals. However, given our focus on the K–Pg transition and not the ancestral condition of the earliest crown mammals, we elected to restrict our analyses to taxa whose nesting and residence ecology can be scored consistently and systematically.

Compared with other major crown mammalian subclades, we infer early arboreal substrate use in Primatomorpha (Meredith et al., 2011) and Euarchonta (Upham et al., 2019), implying either a rapid adoption of arboreality as forests recovered following the K–Pg transition, or retention of at least facultative arboreality across the extinction event. Although relevant fossil data are limited, we can evaluate the primatomorphan and euarchontan fossil record in order to draw inferences about the relative likelihood of these alternative scenarios. The oldest total group primates known from the fossil record (Chester & Sargis, 2020), including the stem primates *Purgatorius* and *Torrejonia* and the crown primate *Teilhardina,* date to within approximately 10 million years following the K–Pg transition (Chester et al., 2015, 2019; Morse et al., 2019). These fossils provide insight into ancestral primate habits in the aftermath of the end‐Cretaceous mass extinction. From studies of postcranial morphology, *Purgatorius* and other stem primates like *Torrejonia* are reconstructed as having been specialized for arboreal habits (Chester et al., 2015, 2019). As stem primates, this hypothesis is consistent with our inference that primatomorphans (Meredith et al., 2011) or euarchontans (Upham et al., 2019) may have retained a capacity for arboreality through the K–Pg. The inferred arboreal habits of this lineage across the K–Pg boundary are intriguing in light of an apparently strong selective filter against arboreal birds at this same time (Field et al., 2018), as well as theoretical and paleobotanical evidence suggesting forest devastation on a global scale following the Chicxulub asteroid impact (Tschudy et al., 1984; Vajda et al., 2001). Although primatomorphans or euarchontans may have retained arboreal habits in hypothetical forested refugia throughout the K–Pg transition, behavioral flexibility and facultative nonarboreality may also have facilitated the survival of arboreally adapted early primatomorphans across the K–Pg. Though extant colugos are specialized gliders and strict herbivores, extant primates have been hypothesized to be resilient in the face of rapid environmental change on account of their sociality, cognition, and dietary and locomotor flexibility (Mekonnen et al., 2018; Morris et al., 2011), and at least some of these and other traits (e.g., omnivory and small body size in the oldest known stem and crown primates (Szalay & Delson, 1979)) may have contributed to the survival of representatives of the primate total group when facing the devastation of forests at the end‐Cretaceous.

There is evidence under some of our models that the early evolutionary history of crown marsupials may have also occurred in an arboreal ecological context (Figure 1; Figures S4, S6, S13, S14, S16, and S18). Our ARD model and in some cases the similar four‐rate model yield an arboreal reconstruction for the most recent common ancestor of crown marsupials (Figure 1; Figures S6, S14, S16, and S18). This inference implies repeated losses of arboreality among marsupials, which would be consistent with the hypothesized retention of plesiomorphic arboreal features in their hands and feet (Bensley, 1901; Haines, 1958; Szalay, 1984). Marsupials suffered some of the greatest diversity loss and longest recovery times in the wake of the K–Pg compared with other boundary‐crossing mammalian groups (Pires et al., 2018), and we infer a signal of consistent arboreality among several marsupial lineages near the K–Pg boundary. This is congruent with the earliest known post‐K–Pg metatherian skeletons from the early Paleocene of Bolivia, which have been reconstructed as scansorial, with *Mayulestes* inferred to be more specialized for arboreality than *Pucadelphys* (Argot, 2003).

Notably, although the fossil record of stem‐group bats (Chiroptera) is sparse, the ancestors of crown bats may have been arboreal before they acquired a capacity for powered flight (Bishop, 2008; Gunnell & Simmons, 2005). However, our results reconstruct much of the chiropteran total group as predominantly nonarboreal through most of the Paleocene and extending back into the Cretaceous (Figure 2) (or, in the case of the ARD and four‐rate models, potentially semi‐arboreal). This is probably a result of the strict application of our character state definitions, where most extant bats were classified as nonarboreal. Many bat species are cave‐roosting—thus, they are classified as nonarboreal or semi‐arboreal in our analyses, highlighting the fact that our classification of “nonarboreality” does not necessarily imply a predominantly ground‐dwelling ecology.

A number of major clades whose extant representatives exhibit arboreality across multiple family‐level subclades (e.g., primatomorphans or euarchontans, marsupials, and xenarthrans) may have retained a capacity for arboreal habits across the K‐Pg boundary and may have already been adapted to exploit arboreal niches relatively quickly as these habitats recovered. By contrast, arboreal latecomers (e.g., dormice, tree squirrels, bats) independently acquired arboreal habits well into the Cenozoic (Figures 1 and 2). In the case of Xenarthra, the earliest known fossil representatives of this group were likely adapted for fossoriality (Gaudin & Croft, 2015), with arboreality in sloths evolving repeatedly and independently throughout the Cenozoic, presumably in response to factors such as diet specialization and predator evasion (Delsuc et al., 2018, 2019). This pattern appears to be reflected in our ASRs: across the majority of our analyses, we infer nonarboreal ecologies for Xenarthra until very shortly after the K–Pg boundary.

As in birds (Field et al., 2018; Mayr, 2016), we hypothesize that nonarboreal habits were associated with increased rates of survivorship among mammals across the K–Pg boundary, consistent with earlier qualitative proposals for enhanced survivorship among burrowing/semi‐aquatic mammals (DeBey & Wilson, 2017; Robertson et al., 2004). Alongside selection against strict arboreality, many mammalian lineages that passed through the K–Pg mass extinction may have been characterized by reduced body size relative to their pre‐extinction antecedents (Lyson et al., 2019); perhaps related to the relationship between body size and total metabolic requirements (Berv & Field, 2018; McNab, 2012), as well as enhanced survivorship among insectivores and omnivores compared with strict carnivores and herbivores (Aberhan et al., 2007; Sheehan & Hansen, 1986). Large‐bodied mammals and dietary specialists appear to have been heavily selected against in the immediate wake of the Chicxulub impact (Grossnickle & Newham, 2016; Lyson et al., 2019; Wilson, 2013). The disparity of multituberculate dentition and body size expanded in the Late Cretaceous (Wilson et al., 2012, Weaver and Wilson 2020), but total mammalian morphological disparity appears to have contracted immediately following the K‐Pg extinction (Wilson, 2013, Grossnickle and Newham 2016). As mammals recovered after the mass extinction and diversified into niches previously occupied by large dinosaurs, maximum mammal body size continued to increase until about 40 mya (Smith et al., 2010).

### 4.2 Analytical assumptions

The evolutionary scenarios proposed here are conditional on the accuracy of the timescale of the extant mammalian radiation estimated in both the Meredith et al. (2011) and Upham et al. (2019) phylogenies. Divergence times estimated with molecular clock models (Bininda‐Emonds et al., 2012; Meredith et al., 2011; Wray, 2002) may greatly exceed estimates of clade ages derived from fossil evidence (Forest, 2009; O'Leary et al., 2013; Wible et al., 2007), and our understanding of the factors underlying this incongruence is improving (Brochu et al., 2004; Field, Berv, et al., 2020; Hillis, 1987; Larson, 1998; Novacek, 1993; O'Leary et al., 2013; Patterson, 1987; Phillips, 2016; Springer, 2004; Springer et al., 2003, 2013). In Xenarthra, divergence time analyses from molecular clock models have yielded estimates for the age of the crown clade exceeding 70 Ma (Bininda‐Emonds et al., 2007), whereas the oldest crown group xenarthran fossils are approximately 59 Ma (O'Leary et al., 2013). Such discrepancies, which span the K–Pg boundary (ca. 66.02 Ma; Clyde et al., 2016), indicate uncertainty regarding the “true” age of important nodes across the mammalian tree of life. This uncertainty is especially relevant to our reconstructions of crown Primatomorpha, for which molecular divergence time analyses frequently estimate a Late Cretaceous origin (Bininda‐Emonds et al., 2007; Janečka et al., 2007; Meredith et al., 2011) and likewise for Euarchonta (Janečka et al., 2007; Upham et al., 2019). At present, the oldest known total‐clade euarchontan—the arboreal stem primate *Purgatorius*—appears shortly after the K–Pg boundary, ca. 65.9 MYA (Wilson Mantilla et al., 2021). Thus, direct fossil evidence bearing on whether arboreality was retained across the K–Pg boundary in euarchontans or primatomorphans is lacking. If the “true” node age is younger than the K‐Pg boundary, it would imply that arboreality may have emerged post‐extinction in Euarchonta or Primatomorpha, rather than arising beforehand and being maintained across the extinction horizon. Lastly, we note that the taxon sample in the present analysis, which is mostly restricted to mammalian family‐level clades, could also have introduced some bias into our analysis, though it is difficult to quantify how this might affect our results a priori (primarily, we expect transition rates to be under‐estimated under the present taxon sampling strategy). Mammalian families that exhibit a range of substrate preferences across extant species‐level diversity are necessarily represented in our consensus trees by only a single taxon; 36% of such families were scored as arboreal. Therefore, further exploration of these questions in the context of an expanded taxon sample would provide a fruitful direction for future research.

## 5 CONCLUSIONS

The short‐term ecological ramifications of the K–Pg mass extinction are difficult to fully assess from our vantage point 66 million years later, but it is increasingly clear that the evolutionary trajectories of arboreal lineages across the vertebrate tree of life were deeply impacted by this mass extinction event (Feng et al., 2017; Field et al., 2018; Vajda et al., 2001). Direct fossil evidence of mammalian ecological habits from the latest Cretaceous and Paleocene will be needed to further test the patterns of mammalian ecological selectivity inferred here. The Late Cretaceous *Deccanolestes* has been interpreted as arboreal, as have its close relatives (the Paleocene adapisoriculids), providing a compelling example of continuous arboreality among noneuarchontan mammals that survived across the K–Pg boundary (Goswami et al., 2011). Although some Late Cretaceous multituberculates have also been proposed to have been arboreal based on isolated fragmentary humeri (DeBey & Wilson, 2017), inferences based on the most complete skeletal material support Late Cretaceous forms as predominantly ground dwelling or fossorial (Kielan‐Jaworowska, 1989; Kielan‐Jaworowska & Gambaryan, 1994; Weaver et al., 2021), and some Paleocene taxa as arboreal (Krause & Jenkins, 1983), suggesting survival of predominantly nonarboreal multituberculates across the K–Pg with postextinction transitions to arboreality.

Inferences of mammalian ecological evolution will continue to be refined in light of ongoing improvements in our understanding of mammalian phylogeny, divergence times, and the fossil record (Grossnickle et al., 2019; Halliday & Goswami, 2016b; Meredith et al., 2011; O'Leary et al., 2013; Phillips, 2016; Upham et al., 2019). Nevertheless, our new results and simulations are consistent with the hypothesis that the K–Pg transition was a fundamental agent driving ecological shifts in the evolutionary history of Mammalia. The phylogeny of crown group mammals appears to retain the selective signature of end‐Cretaceous forest devastation over 66 million years ago, emphasizing the profound degree to which the evolutionary trajectories of extant terrestrial vertebrates were influenced by the K–Pg catastrophe.

## CONFLICT OF INTEREST

The authors declare no conflict of interest.

## AUTHOR CONTRIBUTIONS

**Jonathan J. Hughes:** Conceptualization (equal); data curation (lead); formal analysis (equal); investigation (lead); methodology (equal); project administration (equal); software (equal); validation (equal); visualization (equal); writing—original draft (lead); writing—review and editing (equal). **Jacob S. Berv:** Conceptualization (equal); data curation (equal); formal analysis (lead); investigation (equal); methodology (lead); project administration (equal); software (lead); supervision (equal); validation (equal); visualization (lead); writing—review and editing (equal). **Stephen G. B. Chester:** Supervision (equal); validation (equal); writing—review and editing (equal). **Eric J. Sargis:** Supervision (equal); validation (equal); writing—review and editing (equal). **Daniel J. Field:** Conceptualization (equal); funding acquisition (equal); investigation (supporting); project administration (equal); resources (equal); supervision (equal); writing—review and editing (equal).

## Supporting information

Supplementary Material

## Data Availability

R code will be updated at the author's GitHub repository (https://github.com/jakeberv/mammal_arboreality) and is preserved as a Zenodo archive https://doi.org/10.5281/zenodo.5338540.
