# Supplementary material for: Ecological selectivity and the evolution of mammalian substrate preference across the K–Pg boundary
Source: Ecol Evol. 2021 Oct 11;11(21):14540–54. doi: 10.1002/ece3.8114 (PMC8571592; doi:10.1002/ece3.8114)
Supplement: Supplementary file 1 — Supplementary Material [file ECE3-11-14540-s001.pdf]

Supporting Information

Ecological selectivity and the evolution of mammalian substrate preference across the K–Pg boundary

Jonathan J. Hughes[1,ƛ,*], Jacob S. Berv[1,2,3,ƛ], Stephen G. B. Chester[4,5,6], Eric J. Sargis[7,8,9], Daniel J. Field[10,11*]

1. Department of Ecology & Evolutionary Biology, Cornell University, Ithaca, NY, USA

2. Department of Ecology & Evolutionary Biology, University of Michigan, Ann Arbor, MI, USA

3. University of Michigan Museum of Paleontology, University of Michigan, Ann Arbor, MI, USA

4. Department of Anthropology, Brooklyn College, City University of New York, Brooklyn, NY, USA

5. Department of Anthropology, The Graduate Center, City University of New York, New York, NY, USA

6. New York Consortium in Evolutionary Primatology, New York, NY, USA

7. Department of Anthropology, Yale University, New Haven, CT, USA

8. Divisions of Vertebrate Paleontology and Vertebrate Zoology, Yale Peabody Museum of Natural History, New Haven, CT, USA

9. Yale Institute for Biospheric Studies, New Haven, CT, USA

10. Department of Earth Sciences, University of Cambridge, Cambridge, UK

11. Museum of Zoology, University of Cambridge, UK

[ƛ]Authors contributed equally

*Authors for correspondence: jjh359@cornell.edu; djf70@cam.ac.uk

Table S1.: Taxa that differ between the Meredith et al. (2011) and the Upham et al. (2019) phylogenetic reconstructions. Usually the same taxa were used in both phylogenies. For our comparisons, where the Upham et al. (2019) dataset did not contain the exact same taxon as Meredith et al. (2011) we substituted the closest taxon that could be assigned the same character state. Further, in some instances Meredith et al. 2011) used genes from multiple species in the same family, concatenated to form a single tip in the tree. In these cases we selected one available species from the Upham et al. (2019) dataset to represent this family, so as to keep the taxon sets as phylogenetically equivalent as possible. Other differences are the result of changes in Linnaean names between 2011 and 2019.

**Meredith et al. 2011: taxon**	**Meredith et al. 2011: common name**	**Upham et al. 2019: taxon**	**Upham et al. 2019: common name**
Family Calomyscidae	Mouse-like Hamsters	*Calomyscus bailwardi*	Zagros Mountains Mouse-like Hamster
Canis familiaris	Domesticated Dog	*Canis lupus*	Grey Wolf
Family Megalonychidae	Two-toed Sloths	*Choloepus didactylus*	Linnaeus' Two-toed Sloth
Manis tricuspis	Tree Pangolin	*Phataginus tricuspis*	Tree Pangolin
Family Emballonuridae	Sac-winged Bats	*Emballonura atrata*	Peters's Sheath-tailed Bat
Family Gliridae	Dormice	*Glis glis*	Edible Dormouse
Family Hippopotamidae	Hippos	*Hippopotamus amphibius*	River Hippopotamus
Family Hystricidae	Old World Porcupines	*Hystrix brachyura*	Malayan Porcupine

Family Moschidae	Musk Deer	*Moschus moschiferus*	Siberian Musk Deer
Family Pedetidae	Springhares	*Pedetes capensis*	South African Springhare
Family Leporidae	Rabbits and Hares	*Oryctolagus cuniculus*	European Rabbit
Family Sciuridae	Squirrels	*Tamias striatus*	Eastern Chipmunk

References

Meredith, R. W., J. E. Janečka, J. Gatesy, O. A. Ryder, C. A. Fisher, E. C. Teeling, A. Goodbla, E. Eizirik, T. L. L. Simão, T. Stadler, D. L. Rabosky, R. L. Honeycutt, J. J. Flynn, C. M. Ingram, C. Steiner, T. L. Williams, T. J. Robinson, A. Burk-Herrick, M. Westerman, N. A. Ayoub, M. S. Springer, and W. J. Murphy. 2011. Impacts of the Cretaceous Terrestrial Revolution and KPg extinction on mammal diversification. Science 334:521–524.

Upham, N. S., J. A. Esselstyn, and W. Jetz. 2019. Inferring the mammal tree: Species-level sets of phylogenies for questions in ecology, evolution, and conservation. PLoS Biol. 17:e3000494.

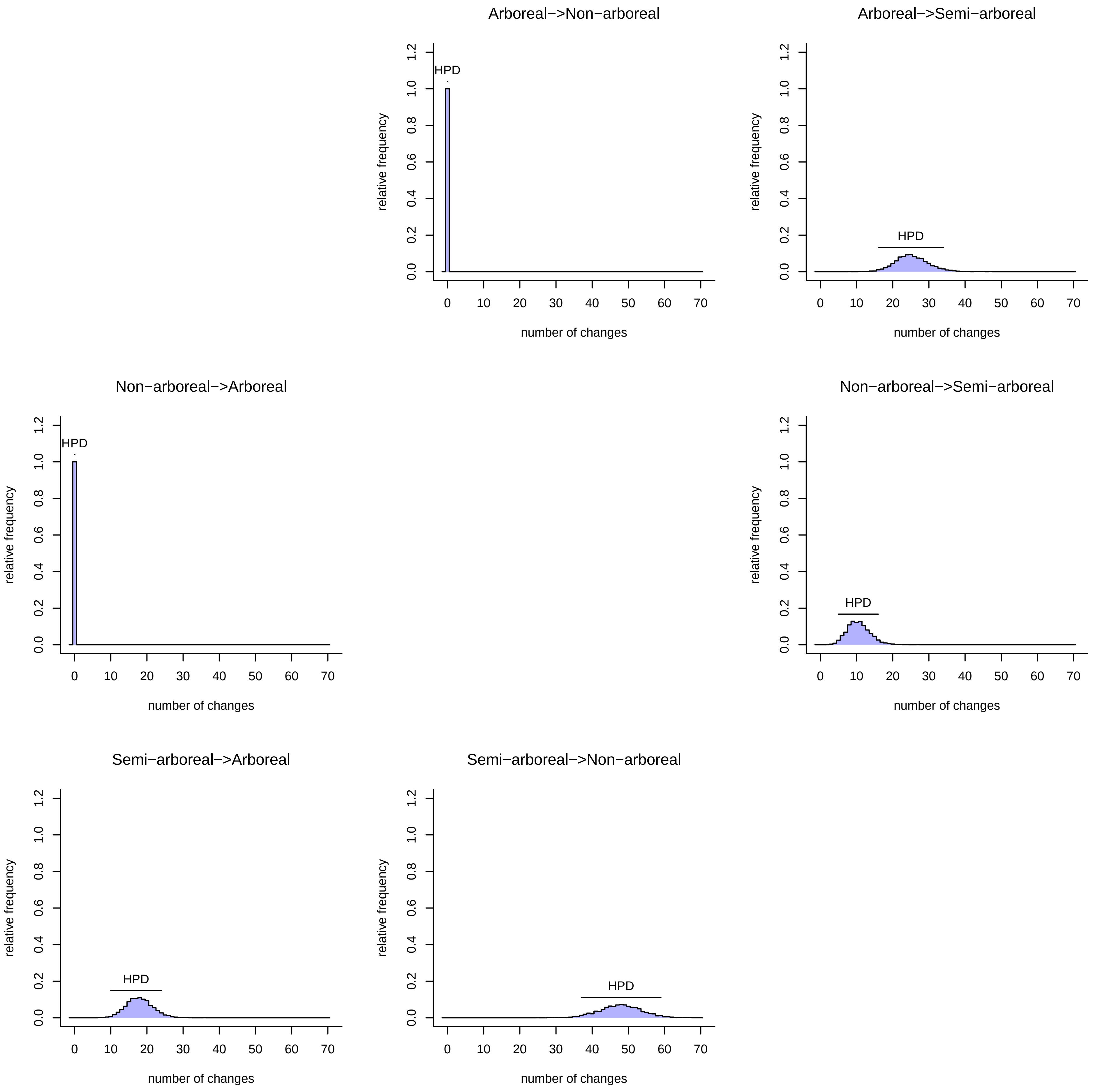
Arboreal−>Non−arboreal
Arboreal−>Semi−arboreal
Non−arboreal−>Arboreal
Non−arboreal−>Semi−arboreal
Semi−arboreal−>Arboreal
Semi−arboreal−>Non−arboreal
HPD
relative frequency
number of changes
0.0
0.2
0.4
0.6
0.8
1.0
1.2
0
10
20
30
40
50
60
70

Figure S1: Transition rate probability distributions from SIMMAP four-rate model applied to the Meredith et al. (2011) topology (Fig. 1).

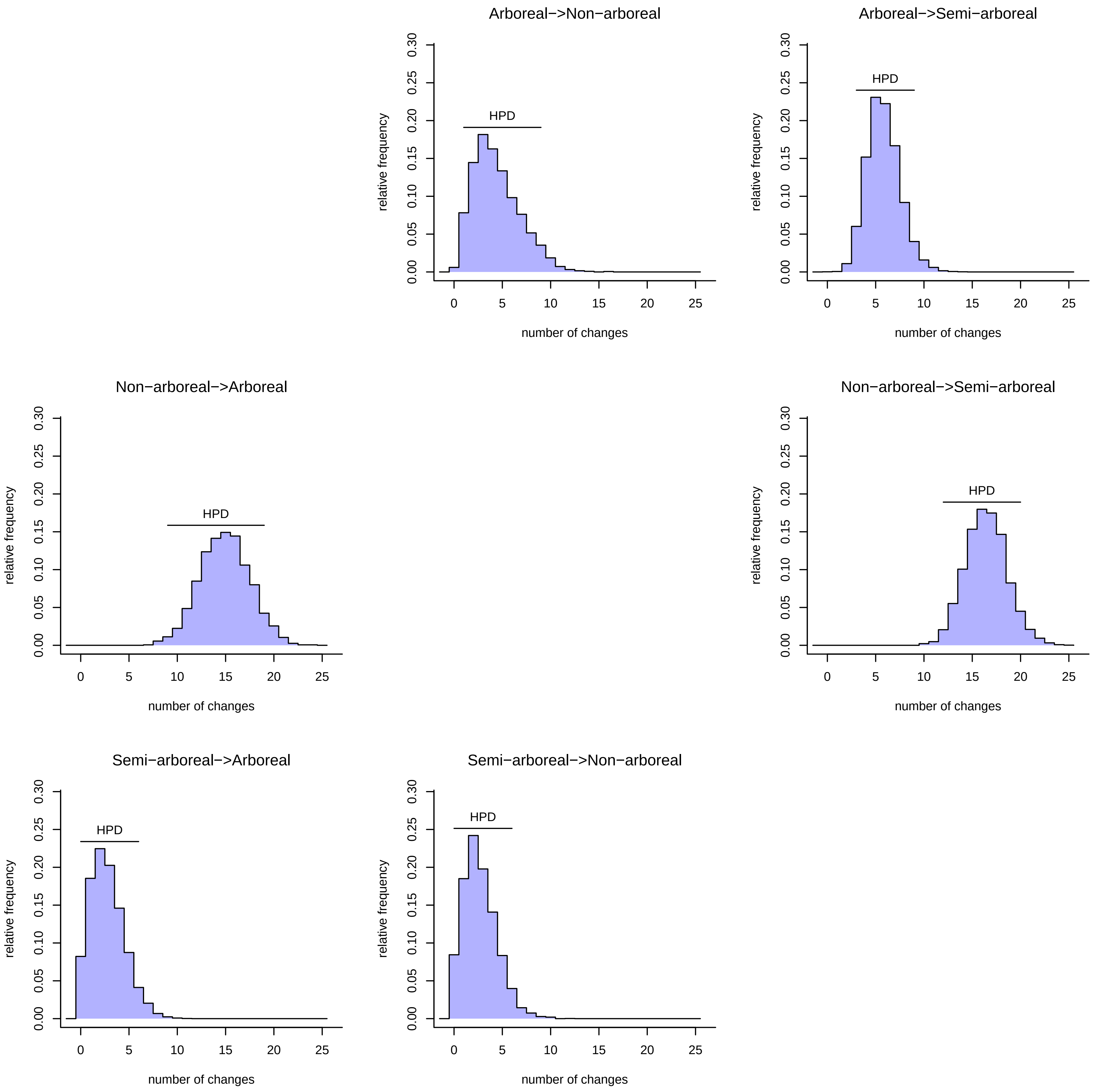
Arboreal−>Non−arboreal
Arboreal−>Semi−arboreal
Non−arboreal−>Arboreal
Non−arboreal−>Semi−arboreal
Semi−arboreal−>Arboreal
Semi−arboreal−>Non−arboreal
HPD
relative frequency
number of changes
0.00 0.05 0.10 0.15 0.20 0.25 0.30
0 5 10 15 20 25

Figure S2: Transition rate probability distributions from SIMMAP two-rate model applied to the Meredith et al. (2011) topology (Fig. S5)

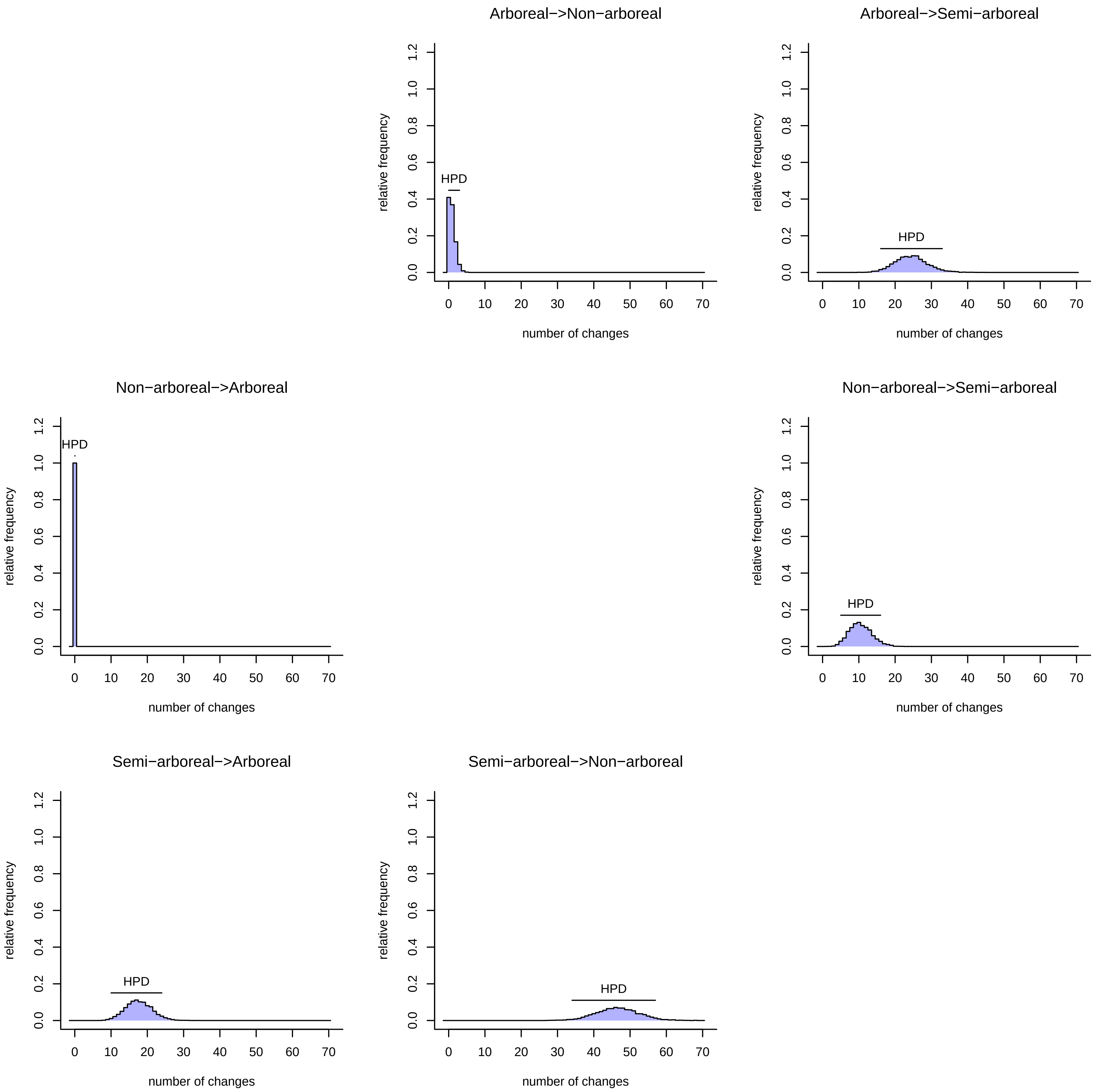
Arboreal−>Non−arboreal
Arboreal−>Semi−arboreal
Non−arboreal−>Arboreal
Non−arboreal−>Semi−arboreal
Semi−arboreal−>Arboreal
Semi−arboreal−>Non−arboreal
HPD
relative frequency
number of changes

Figure S3: Transition rate probability distributions from SIMMAP ARD model applied to the Meredith et al. (2011) topology (Fig. S6).

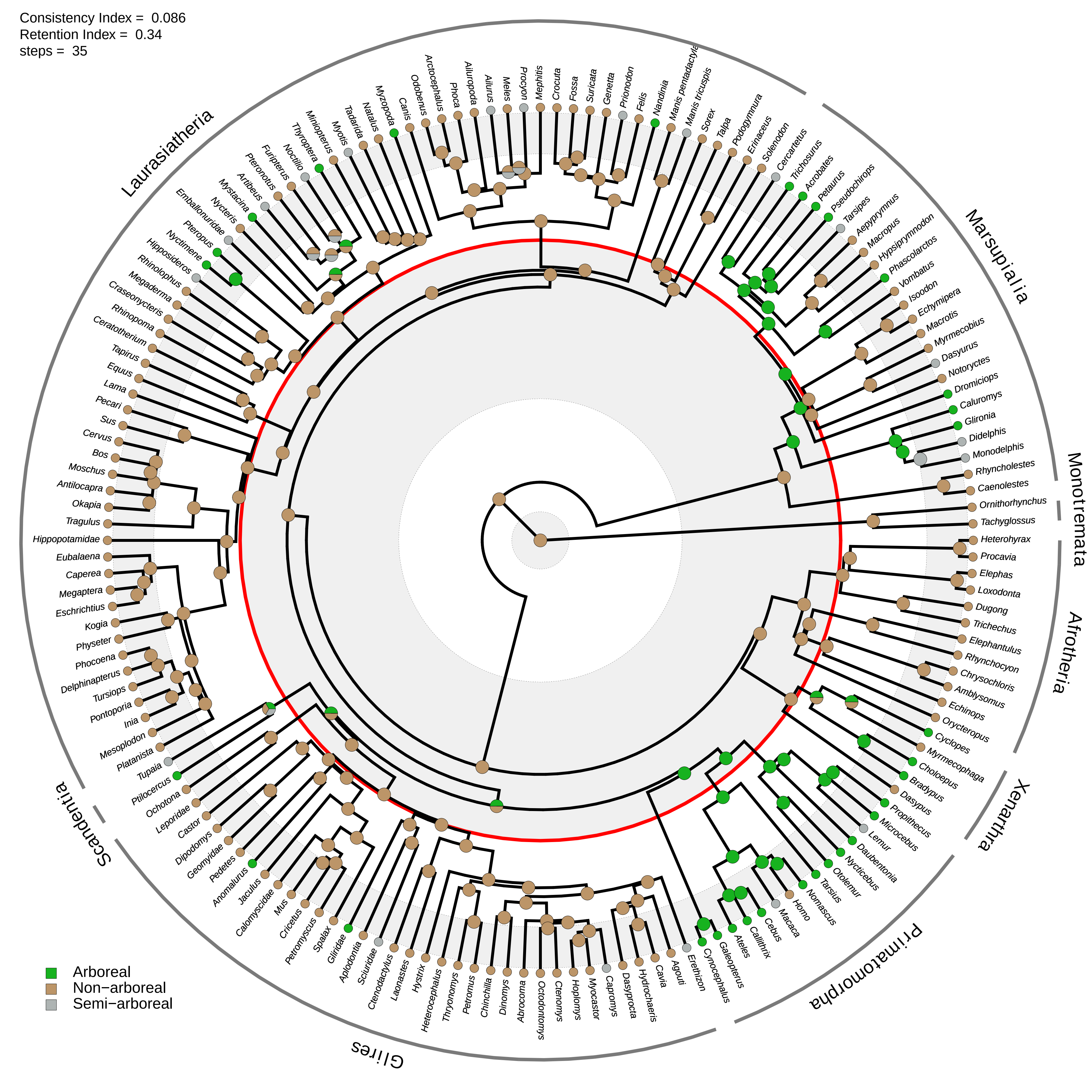
Consistency Index = 0.086
Retention Index = 0.34
steps = 35
Laurasiatheria
Marsupialia
Monotremata
Afrotheria
Xenarthra
Primatomorpha
Glires
Scandentia
Emballonuridae
Pteropus
Nyctimene
Hipposideros
Rhinolophus
Megaderma
Craseonycteris
Rhinopoma
Ceratotherium
Tapirus
Equus
Lama
Pecari
Sus
Cervus
Bos
Moschus
Antilocapra
Okapia
Tragulus
Hippopotamidae
Eubalaena
Caperea
Megaptera
Eschrichtius
Kogia
Physeter
Phocoena
Delphinapterus
Tursiops
Pontoporia
Inia
Mesoplodon
Platanista
Tupaia
Ptilocercus
Ochotona
Leporidae
Castor
Dipodomys
Geomyidae
Pedetes
Anomalurus
Jaculus
Calomyscidae
Mus
Cricetus
Petromyscus
Spalax
Gliridae
Aplodontia
Sciuridae
Ctenodactylus
Laonastes
Hystrix
Heterocephalus
Thryonomys
Petromus
Chinchilla
Dinomys
Abrocoma
Octodontomys
Ctenomys
Hoplomys
Myocastor
Capromys
Dasyprocta
Hydrochaeris
Cavia
Agouti
Erethizon
Cynocephalus
Galeopterus
Ateles
Callithrix
Cebus
Macaca
Homo
Nomascus
Tarsius
Otolemur
Nycticebus
Daubentonia
Lemur
Microcebus
Propithecus
Dasypus
Bradypus
Choloepus
Myrmecophaga
Cyclopes
Orycteropus
Echinops
Amblysomus
Chrysochloris
Rhynchocyon
Elephantulus
Trichechus
Dugong
Loxodonta
Elephas
Procavia
Heterohyrax
Tachyglossus
Ornithorhynchus
Caenolestes
Rhyncholestes
Monodelphis
Didelphis
Glironia
Caluromys
Dromiciops
Notoryctes
Dasyurus
Myrmecobius
Macrotis
Echymipera
Isoodon
Vombatus
Phascolarctos
Hypsiprymnodon
Macropus
Aepyprymnus
Tarsipes
Pseudochirops
Petaurus
Acrobates
Trichosurus
Cercartetus
Solenodon
Erinaceus
Podogymnura
Talpa
Sorex
Manis tricuspis
Manis pentadactyla
Nandinia
Felis
Prionodon
Genetta
Suricata
Fossa
Crocuta
Mephitis
Procyon
Meles
Ailurus
Ailuropoda
Phoca
Arctocephalus
Odobenus
Canis
Myzopoda
Natalus
Tadarida
Myotis
Miniopterus
Thyroptera
Noctilio
Furipterus
Pteronotus
Artibeus
Mystacina
Nycteris
Arboreal
Non−arboreal
Semi−arboreal

Figure S4: Maximum parsimony reconstruction of substrate preference in mammals applied to the Meredith et al. (2011) topology. All mammalian lineages except primatomorphans and most marsupials are reconstructed as non-arboreal across the K–Pg boundary, which is indicated by the red circle. Pie charts indicate most parsimonious character states as identified by the Phangorn package ancestry.pars() (Schliep, 2011).

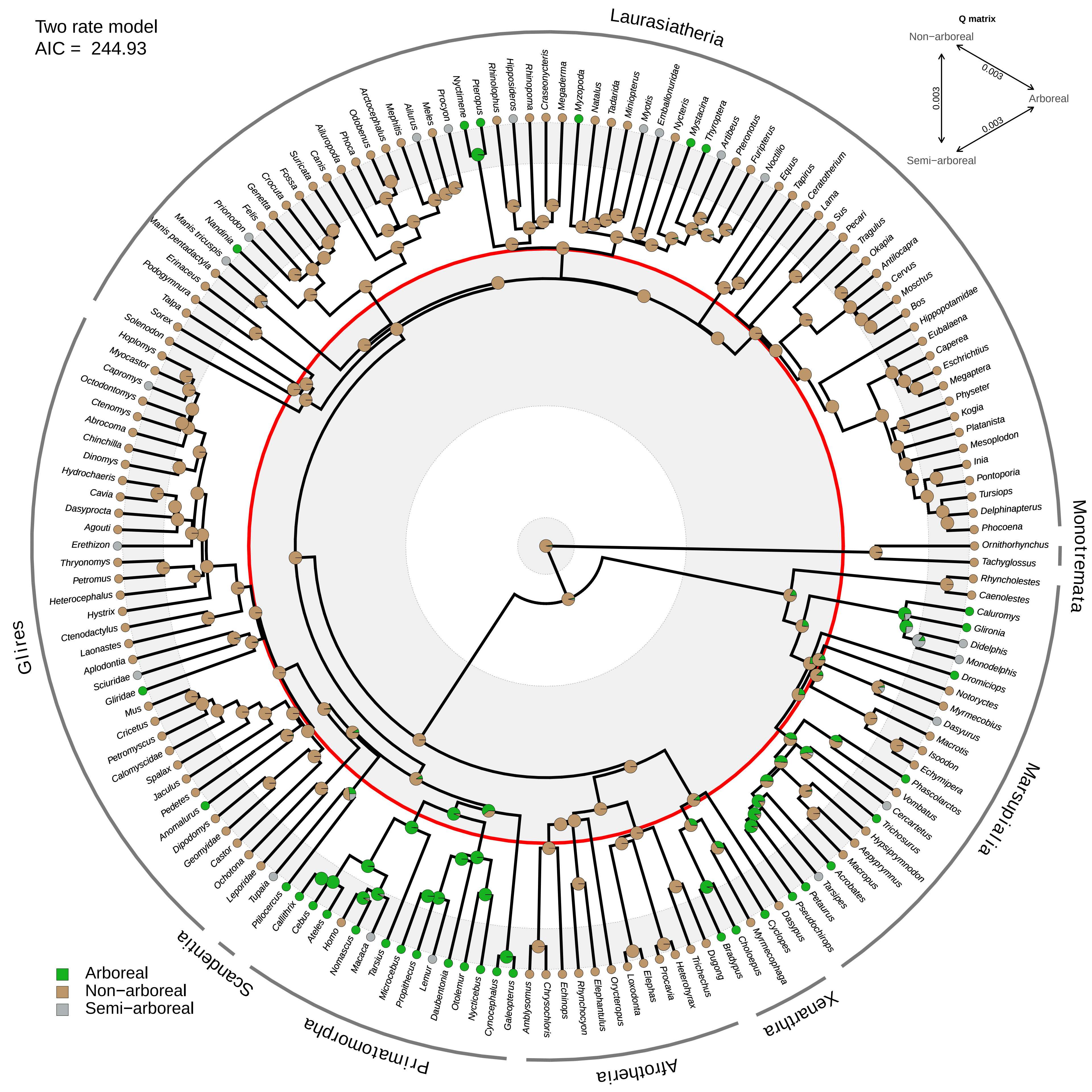

Two rate model
AIC = 244.93
Q matrix
Non−arboreal
Arboreal
Semi−arboreal
0.003
0.003
0.003
Laurasiatheria
Monotremata
Marsupialia
Xenarthra
Afrotheria
Primatomorpha
Scandentia
Glires
Ornithorhynchus
Tachyglossus
Rhyncholestes
Caenolestes
Caluromys
Glironia
Didelphis
Monodelphis
Dromiciops
Notoryctes
Myrmecobius
Dasyurus
Macrotis
Isoodon
Echymipera
Phascolarctos
Vombatus
Cercartetus
Trichosurus
Hypsiprymnodon
Aepyprymnus
Macropus
Acrobates
Tarsipes
Petaurus
Pseudochirops
Dasypus
Cyclopes
Myrmecophaga
Choloepus
Bradypus
Dugong
Trichechus
Heterohyrax
Procavia
Elephas
Loxodonta
Orycteropus
Elephantulus
Rhynchocyon
Echinops
Chrysochloris
Amblysomus
Galeopterus
Cynocephalus
Nycticebus
Otolemur
Daubentonia
Lemur
Propithecus
Microcebus
Tarsius
Macaca
Nomascus
Homo
Ateles
Cebus
Callithrix
Ptilocercus
Tupaia
Leporidae
Ochotona
Castor
Geomyidae
Dipodomys
Anomalurus
Pedetes
Jaculus
Spalax
Calomyscidae
Petromyscus
Cricetus
Mus
Gliridae
Sciuridae
Aplodontia
Laonastes
Ctenodactylus
Hystrix
Heterocephalus
Petromus
Thryonomys
Erethizon
Agouti
Dasyprocta
Cavia
Hydrochaeris
Dinomys
Chinchilla
Abrocoma
Ctenomys
Octodontomys
Capromys
Myocastor
Hoplomys
Solenodon
Sorex
Talpa
Podogymnura
Erinaceus
Manis pentadactyla
Manis tricuspis
Nandinia
Prionodon
Felis
Genetta
Crocuta
Fossa
Suricata
Canis
Ailuropoda
Phoca
Odobenus
Arctocephalus
Mephitis
Ailurus
Meles
Procyon
Nyctimene
Pteropus
Rhinolophus
Hipposideros
Rhinopoma
Craseonycteris
Megaderma
Myzopoda
Natalus
Tadarida
Miniopterus
Myotis
Emballonuridae
Nycteris
Mystacina
Thyroptera
Artibeus
Pteronotus
Furipterus
Noctilio
Equus
Tapirus
Ceratotherium
Lama
Sus
Pecari
Tragulus
Okapia
Antilocapra
Cervus
Moschus
Bos
Hippopotamidae
Eubalaena
Caperea
Eschrichtius
Megaptera
Physeter
Kogia
Platanista
Mesoplodon
Inia
Pontoporia
Tursiops
Delphinapterus
Phocoena
Arboreal
Non−arboreal
Semi−arboreal

Figure S5: Bayesian ancestral state reconstruction of substrate preference in mammals. Pie charts indicate posterior probabilities of character states from SIMMAP under our two-rate model. Topology is from Meredith et al. (2011). The red circle indicates the K–Pg boundary. Primatomorpha is recovered as arboreal across this boundary, and all other mammalian clades are assigned as non-arboreal across the K–Pg boundary.

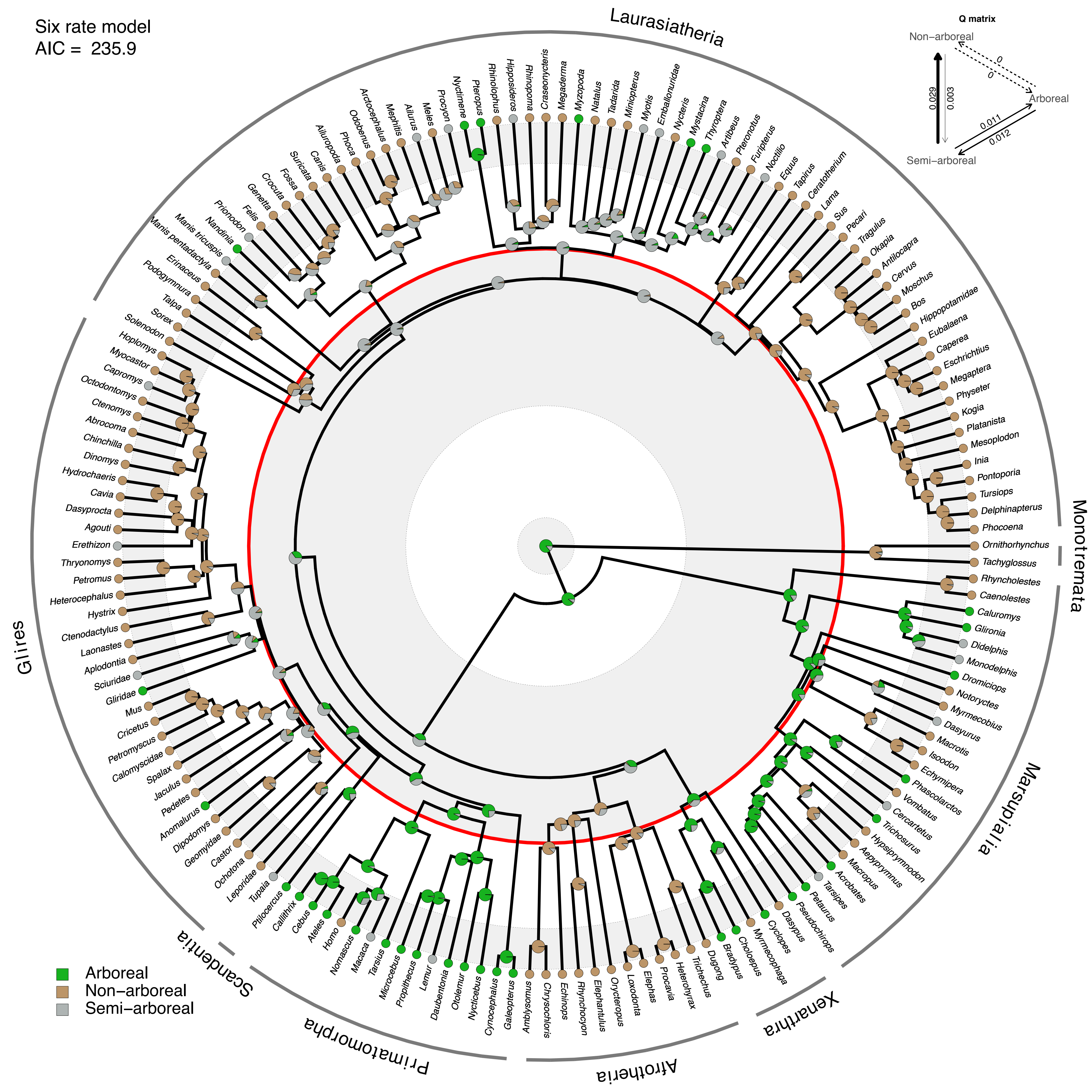

Six rate model
AIC = 235.9
Q matrix
Non–arboreal
Arboreal
Semi–arboreal
0
0
0.029
0.003
0.011
0.012
Laurasiatheria
Monotremata
Marsupialia
Xenarthra
Afrotheria
Primatomorpha
Scandentia
Glires
Arboreal
Non–arboreal
Semi–arboreal
Prionodon
Felis
Genetta
Crocuta
Fossa
Suricata
Canis
Ailuropoda
Phoca
Odobenus
Arctocephalus
Mephitis
Ailurus
Meles
Procyon
Nyctimene
Pteropus
Rhinolophus
Hipposideros
Rhinopoma
Craseonycteris
Megaderma
Myzopoda
Natalus
Tadarida
Miniopterus
Myotis
Emballonuridae
Nycteris
Mystacina
Thyroptera
Artibeus
Pteronotus
Furipterus
Noctilio
Equus
Tapirus
Ceratotherium
Lama
Sus
Pecari
Tragulus
Okapia
Antilocapra
Cervus
Moschus
Bos
Hippopotamidae
Eubalaena
Caperea
Eschrichtius
Megaptera
Physeter
Kogia
Platanista
Mesoplodon
Inia
Pontoporia
Tursiops
Delphinapterus
Phocoena
Ornithorhynchus
Tachyglossus
Rhyncholestes
Caenolestes
Caluromys
Glironia
Didelphis
Monodelphis
Dromiciops
Notoryctes
Myrmecobius
Dasyurus
Macrotis
Isoodon
Echymipera
Phascolarctos
Vombatus
Cercartetus
Trichosurus
Hypsiprymnodon
Aepyprymnus
Macropus
Acrobates
Tarsipes
Petaurus
Pseudochirops
Dasypus
Cyclopes
Myrmecophaga
Choloepus
Bradypus
Dugong
Trichechus
Heterohyrax
Procavia
Elephas
Loxodonta
Orycteropus
Elephantulus
Rhynchocyon
Echinops
Chrysochloris
Amblysomus
Galeopterus
Cynocephalus
Nycticebus
Otolemur
Daubentonia
Lemur
Propithecus
Microcebus
Tarsius
Macaca
Nomascus
Homo
Ateles
Cebus
Callithrix
Ptilocercus
Tupaia
Leporidae
Ochotona
Castor
Geomyidae
Dipodomys
Anomalurus
Pedetes
Jaculus
Spalax
Calomyscidae
Petromyscus
Cricetus
Mus
Gliridae
Sciuridae
Aplodontia
Laonastes
Ctenodactylus
Hystrix
Heterocephalus
Petromus
Thryonomys
Erethizon
Agouti
Dasyprocta
Cavia
Hydrochaeris
Dinomys
Chinchilla
Abrocoma
Ctenomys
Octodontomys
Capromys
Myocastor
Hoplomys
Solenodon
Sorex
Talpa
Podogymnura
Erinaceus
Manis pentadactyla
Manis tricuspis
Nandinia

Figure S6: Bayesian ancestral state reconstruction of substrate preference in mammals applied to the Meredith et al. (2011) topology. Pie charts indicate posterior probabilities of character states from SIMMAP under our ARD model. The red circle indicates the K–Pg boundary. This model finds the most support for arboreal lineages across the boundary. The majority of circum-K-Pg nodes are assigned as semi-arboreal, while Primatomorpha, Scandentia, and Marsupialia, are all identified as arboreal at the K–Pg. Xenarthra is marginally more frequently identified as arboreal than semi-arboreal at the node immediately prior to the K–Pg.

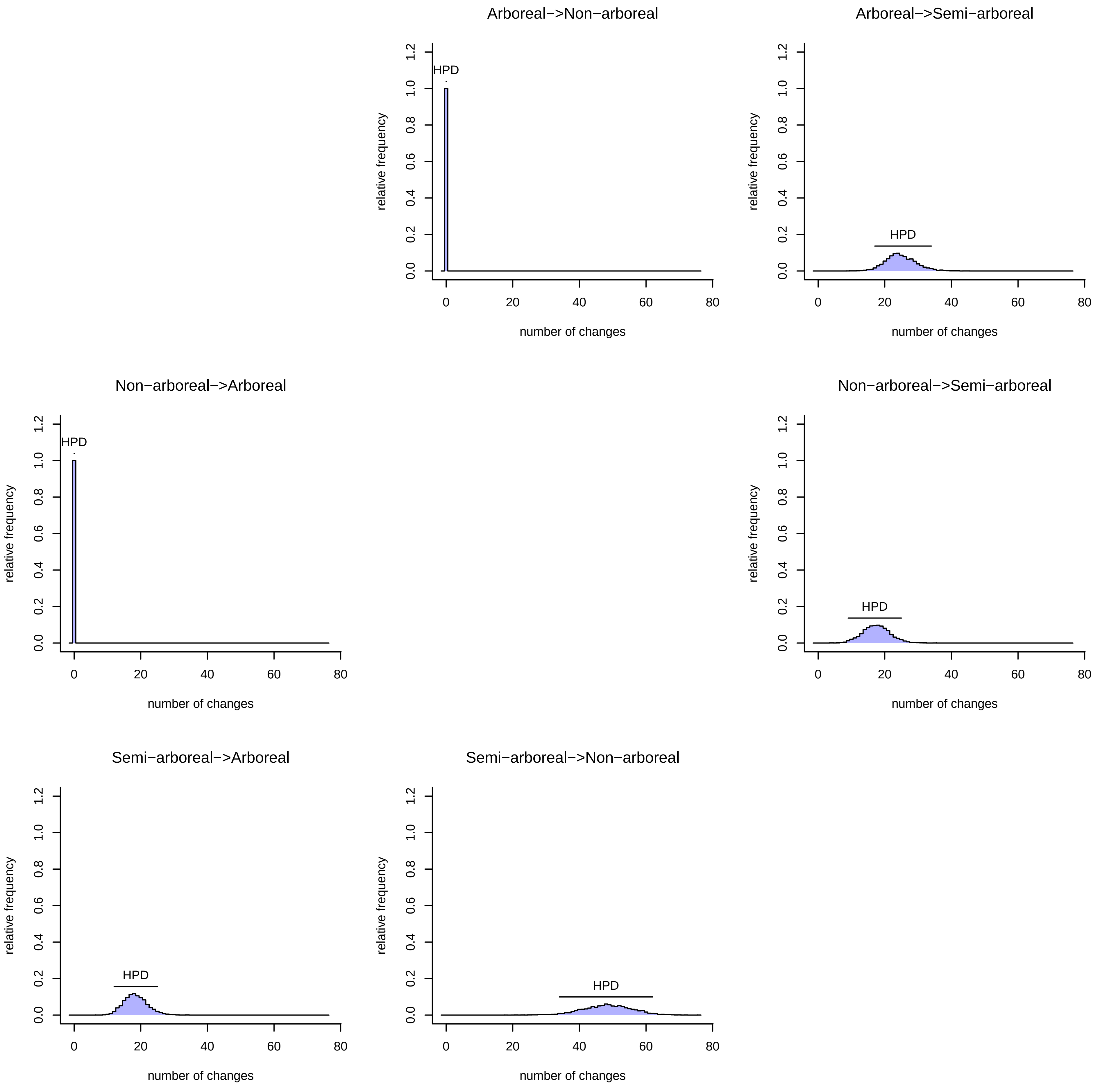

Arboreal−>Non−arboreal
Arboreal−>Semi−arboreal
Non−arboreal−>Arboreal
Non−arboreal−>Semi−arboreal
Semi−arboreal−>Arboreal
Semi−arboreal−>Non−arboreal
HPD
relative frequency
number of changes
0.0
0.2
0.4
0.6
0.8
1.0
1.2
0
20
40
60
80

Figure S7: Transition rate probability distributions from SIMMAP four-rate model applied to the Upham et al. (2011) consensus topology (Fig. S14).

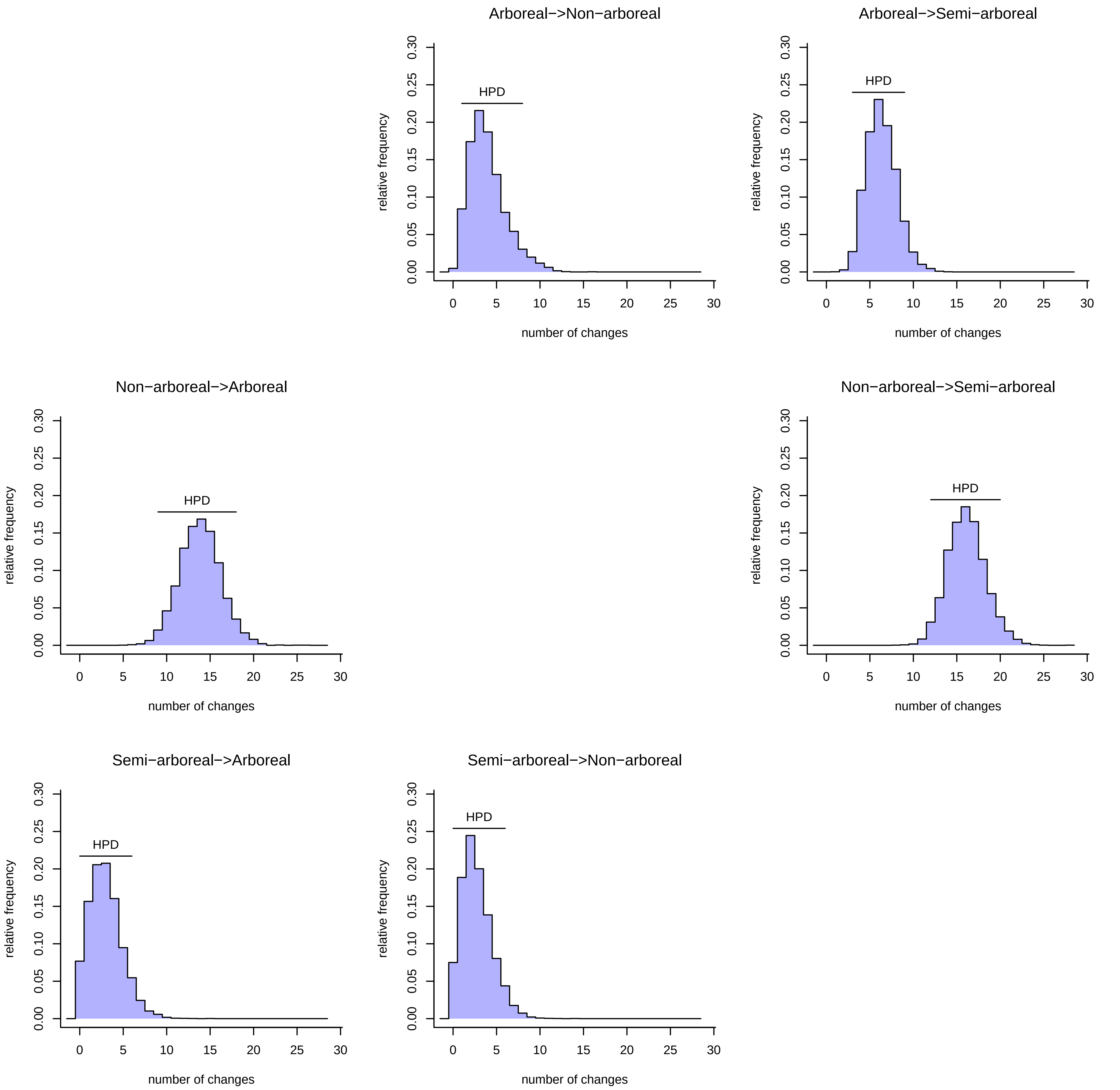

Arboreal−>Non−arboreal
Arboreal−>Semi−arboreal
Non−arboreal−>Arboreal
Non−arboreal−>Semi−arboreal
Semi−arboreal−>Arboreal
Semi−arboreal−>Non−arboreal
HPD
relative frequency
number of changes
0.00 0.05 0.10 0.15 0.20 0.25 0.30
0 5 10 15 20 25 30

Figure S8: Transition rate probability distributions from SIMMAP two-rate model applied to the Upham et al. (2011) consensus topology (Fig. S15).

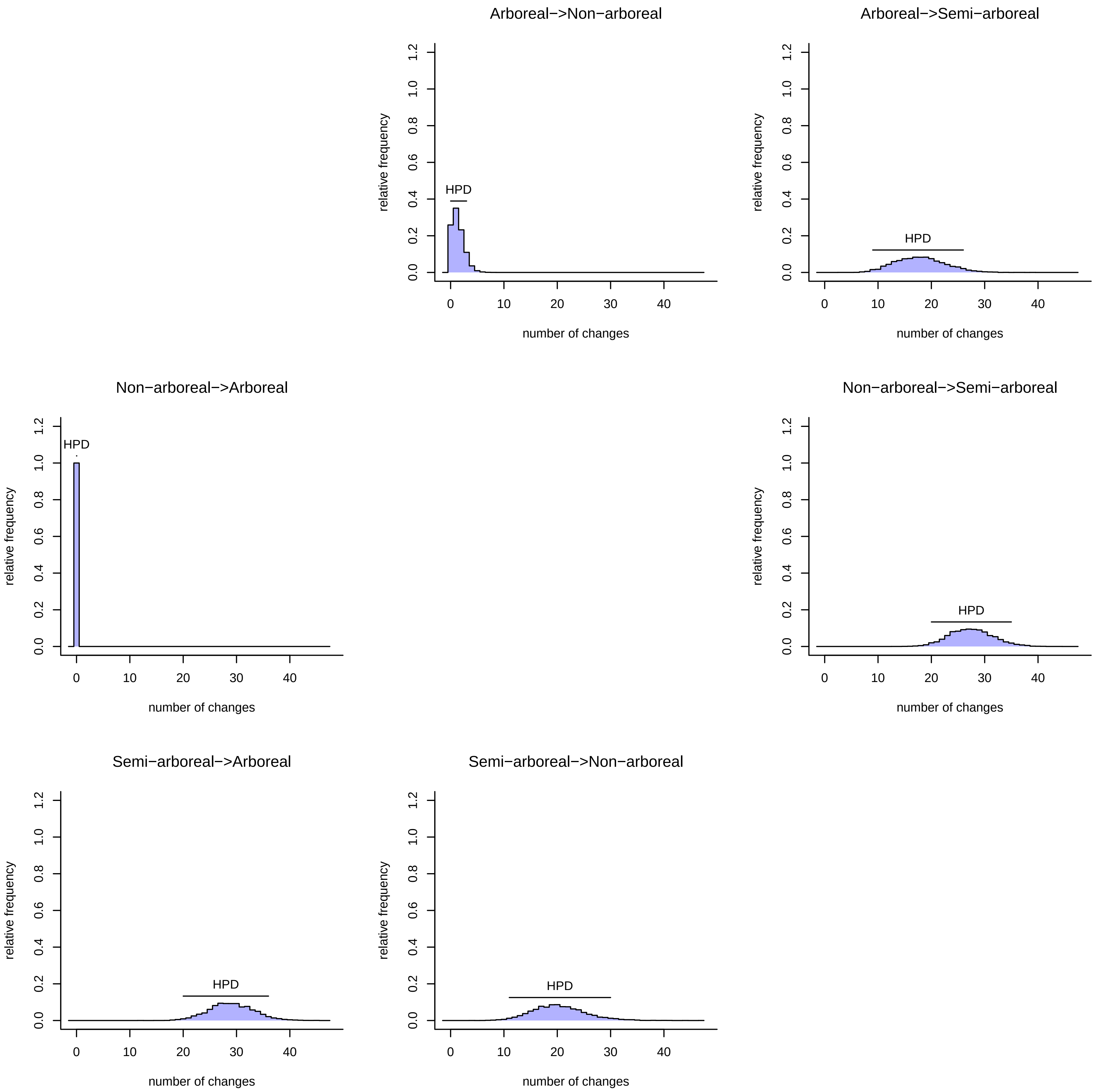
Arboreal−>Non−arboreal
Arboreal−>Semi−arboreal
Non−arboreal−>Arboreal
Non−arboreal−>Semi−arboreal
Semi−arboreal−>Arboreal
Semi−arboreal−>Non−arboreal
HPD
relative frequency
number of changes
0.0
0.2
0.4
0.6
0.8
1.0
1.2
0
10
20
30
40

Figure S9: Transition rate probability distributions from SIMMAP ARD model applied to the Upham et al. (2011) consensus topology (Fig. S16).

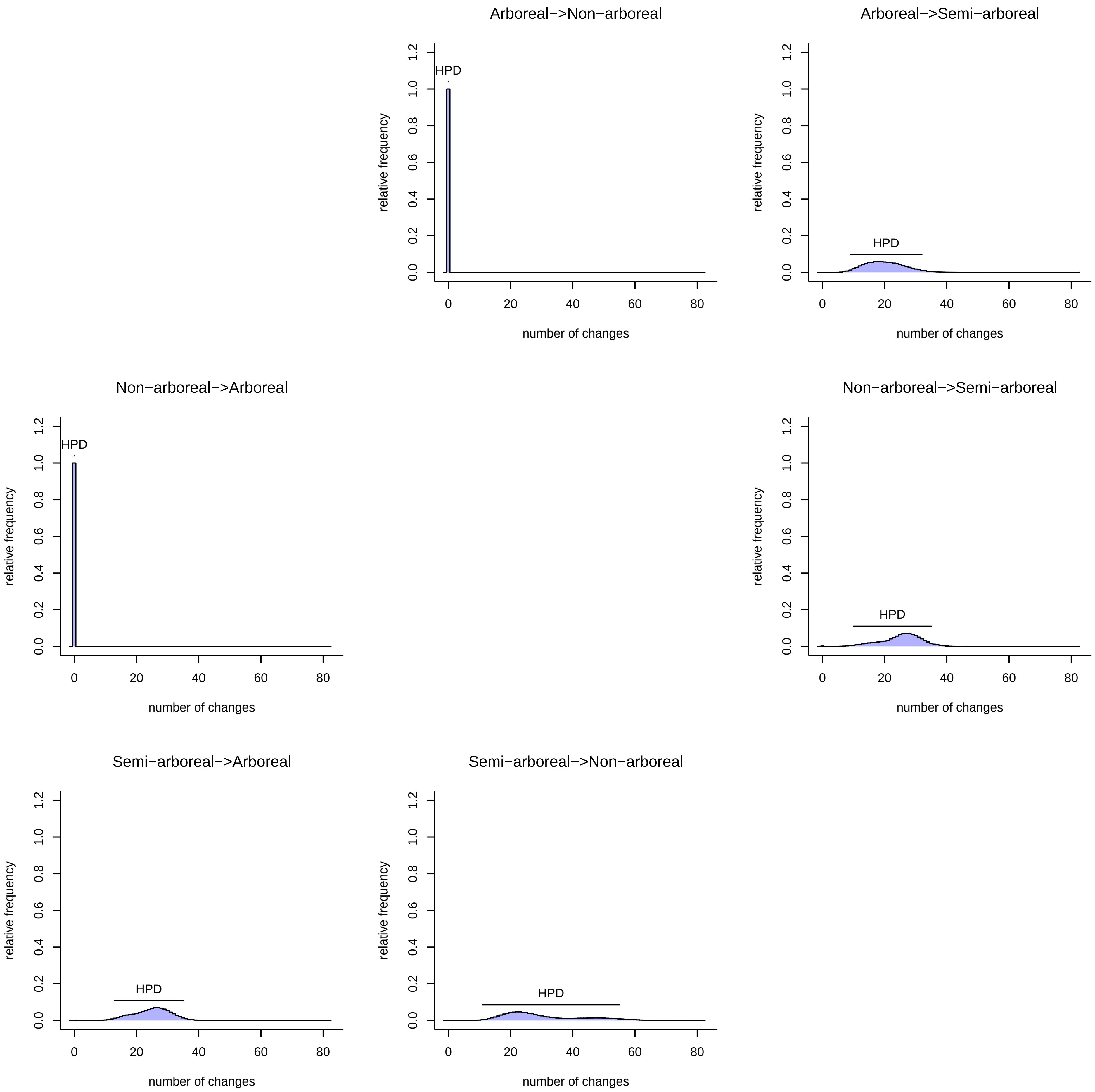
Arboreal−>Non−arboreal
Arboreal−>Semi−arboreal
Non−arboreal−>Arboreal
Non−arboreal−>Semi−arboreal
Semi−arboreal−>Arboreal
Semi−arboreal−>Non−arboreal
HPD
relative frequency
number of changes
0.0
0.2
0.4
0.6
0.8
1.0
1.2
0
20
40
60
80

Figure S10: Transition rate probability distributions from SIMMAP four-rate model applied to the Upham et al. (2011) posterior topologies (Fig. 2).

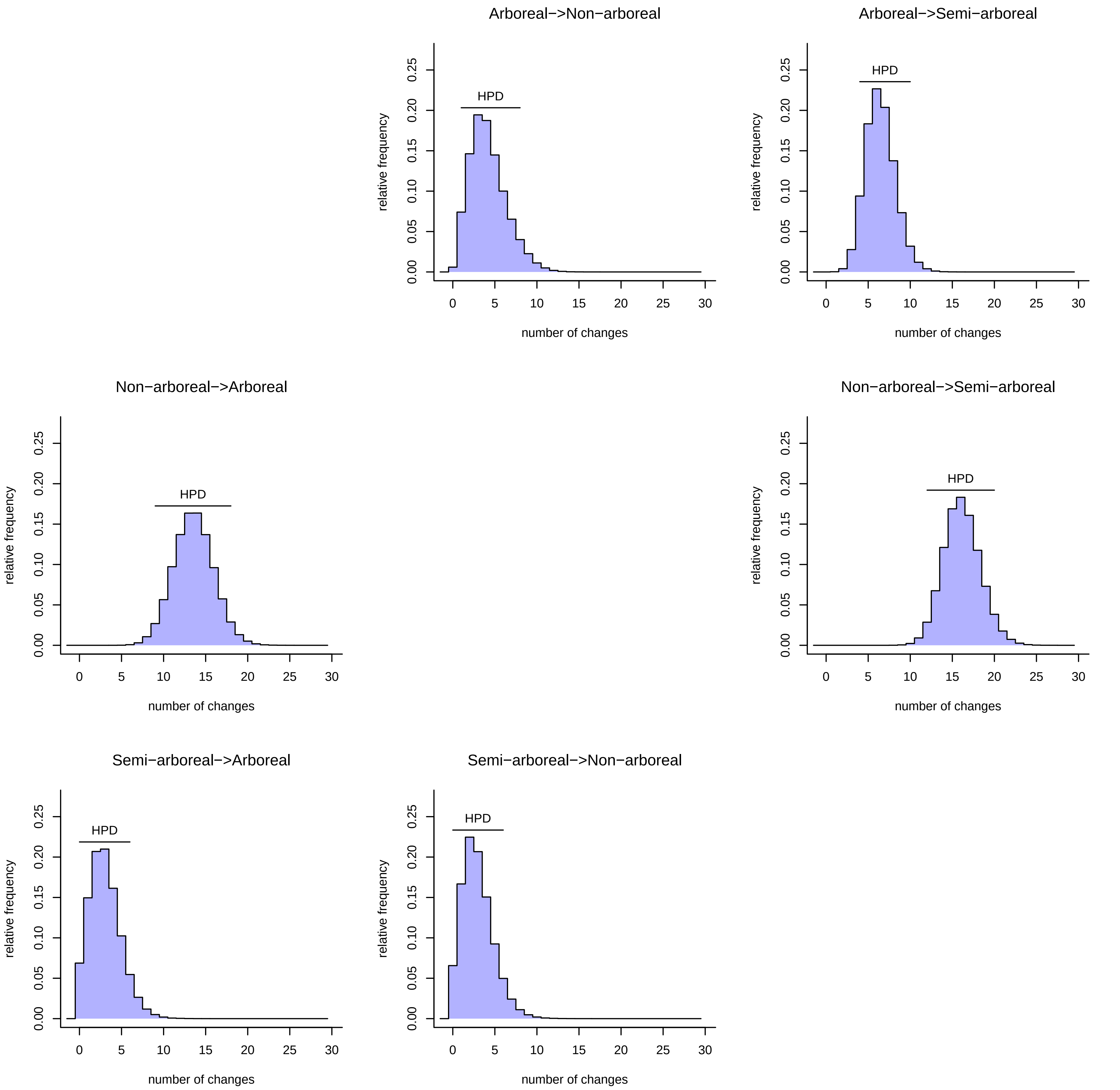
Arboreal−>Non−arboreal
Arboreal−>Semi−arboreal
Non−arboreal−>Arboreal
Non−arboreal−>Semi−arboreal
Semi−arboreal−>Arboreal
Semi−arboreal−>Non−arboreal
HPD
relative frequency
number of changes
0.00
0.05
0.10
0.15
0.20
0.25
0
5
10
15
20
25
30

Figure S11: Transition rate probability distributions from SIMMAP two-rate model applied to the Upham et al. (2011) posterior topologies (Fig. S17).

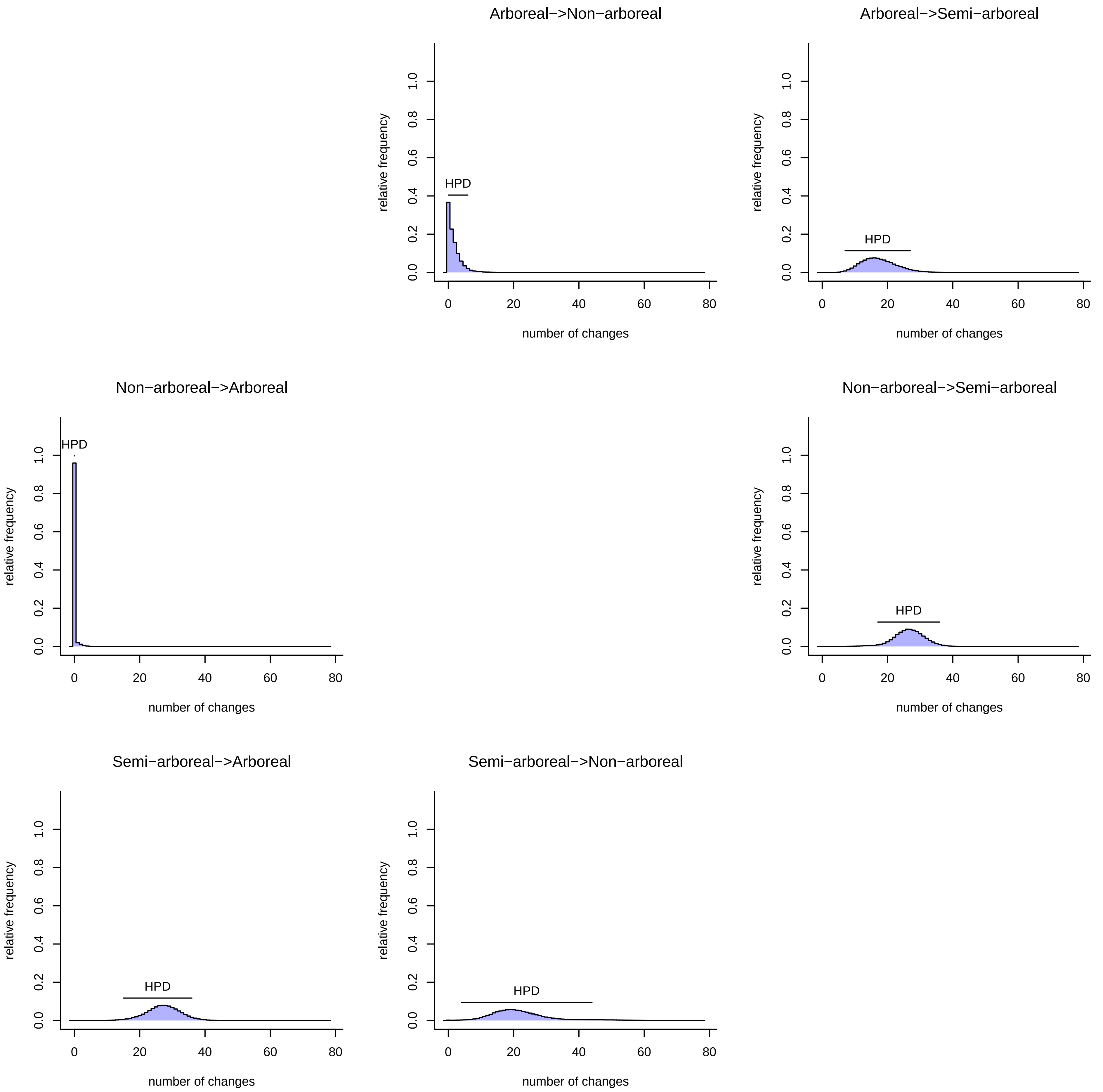
Arboreal−>Non−arboreal
Arboreal−>Semi−arboreal
Non−arboreal−>Arboreal
Non−arboreal−>Semi−arboreal
Semi−arboreal−>Arboreal
Semi−arboreal−>Non−arboreal
HPD
relative frequency
number of changes
0.0
0.2
0.4
0.6
0.8
1.0
0
20
40
60
80

Figure S12: Transition rate probability distributions from SIMMAP ARD model applied to the Upham et al. (2011) posterior topologies (Fig. S18).

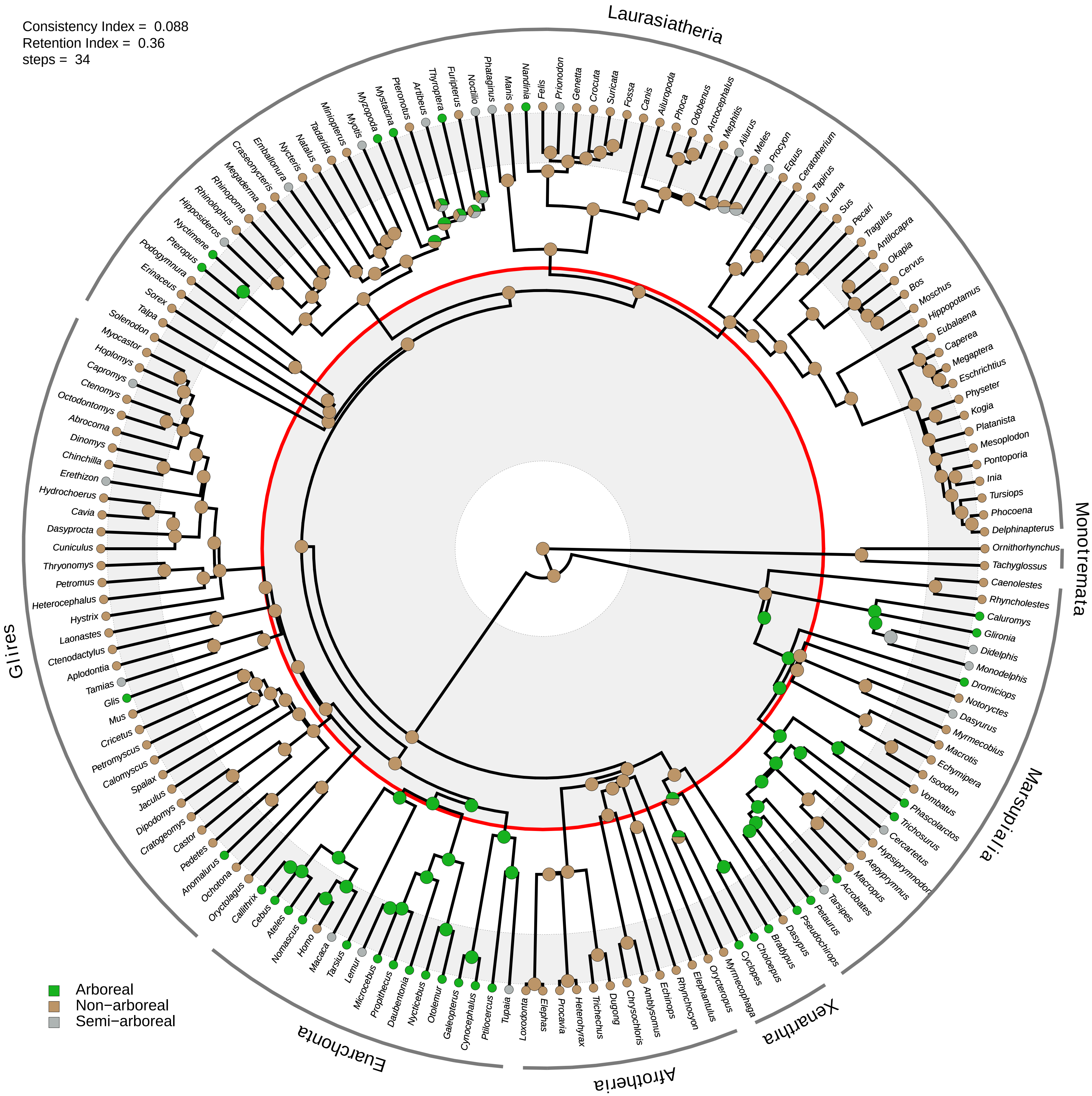
Consistency Index = 0.088
Retention Index = 0.36
steps = 34
Laurasiatheria
Monotremata
Marsupialia
Xenarthra
Afrotheria
Euarchonta
Glires
Arboreal
Non−arboreal
Semi−arboreal
Ornithorhynchus
Tachyglossus
Caenolestes
Rhyncholestes
Caluromys
Glironia
Didelphis
Monodelphis
Dromiciops
Notoryctes
Dasyurus
Myrmecobius
Macrotis
Echymipera
Isoodon
Vombatus
Phascolarctos
Trichosurus
Cercartetus
Hypsiprymnodon
Aepyprymnus
Macropus
Acrobates
Tarsipes
Petaurus
Pseudochirops
Dasypus
Bradypus
Choloepus
Cyclopes
Myrmecophaga
Orycteropus
Elephantulus
Rhynchocyon
Echinops
Amblysomus
Chrysochloris
Dugong
Trichechus
Heterohyrax
Procavia
Elephas
Loxodonta
Tupaia
Ptilocercus
Cynocephalus
Galeopterus
Otolemur
Nycticebus
Daubentonia
Propithecus
Microcebus
Lemur
Tarsius
Macaca
Homo
Nomascus
Ateles
Cebus
Callithrix
Oryctolagus
Ochotona
Anomalurus
Pedetes
Castor
Cratogeomys
Dipodomys
Jaculus
Spalax
Calomyscus
Petromyscus
Cricetus
Mus
Glis
Tamias
Aplodontia
Ctenodactylus
Laonastes
Hystrix
Heterocephalus
Petromus
Thryonomys
Cuniculus
Dasyprocta
Cavia
Hydrochoerus
Erethizon
Chinchilla
Dinomys
Abrocoma
Octodontomys
Ctenomys
Capromys
Hoplomys
Myocastor
Solenodon
Talpa
Sorex
Erinaceus
Podogymnura
Pteropus
Nyctimene
Hipposideros
Rhinolophus
Rhinopoma
Megaderma
Craseonycteris
Emballonura
Nycteris
Natalus
Tadarida
Miniopterus
Myotis
Myzopoda
Mystacina
Pteronotus
Artibeus
Thyroptera
Furipterus
Noctilio
Phataginus
Manis
Nandinia
Felis
Prionodon
Genetta
Crocuta
Suricata
Fossa
Canis
Ailuropoda
Phoca
Odobenus
Arctocephalus
Mephitis
Ailurus
Meles
Procyon
Equus
Ceratotherium
Tapirus
Lama
Sus
Pecari
Tragulus
Antilocapra
Okapia
Cervus
Bos
Moschus
Hippopotamus
Eubalaena
Caperea
Megaptera
Eschrichtius
Physeter
Kogia
Platanista
Mesoplodon
Pontoporia
Inia
Tursiops
Phocoena
Delphinapterus

Figure S13: Maximum parsimony reconstruction of substrate preference in mammals applied to the Upham et al. (2019) consensus topology. All mammalian lineages except euarchontans and most marsupials are reconstructed as non-arboreal across the K–Pg boundary, which is indicated by the red circle. Pie charts indicate most parsimonious character states as identified by the Phangorn package ancestry.pars() (Schliep, 2011).

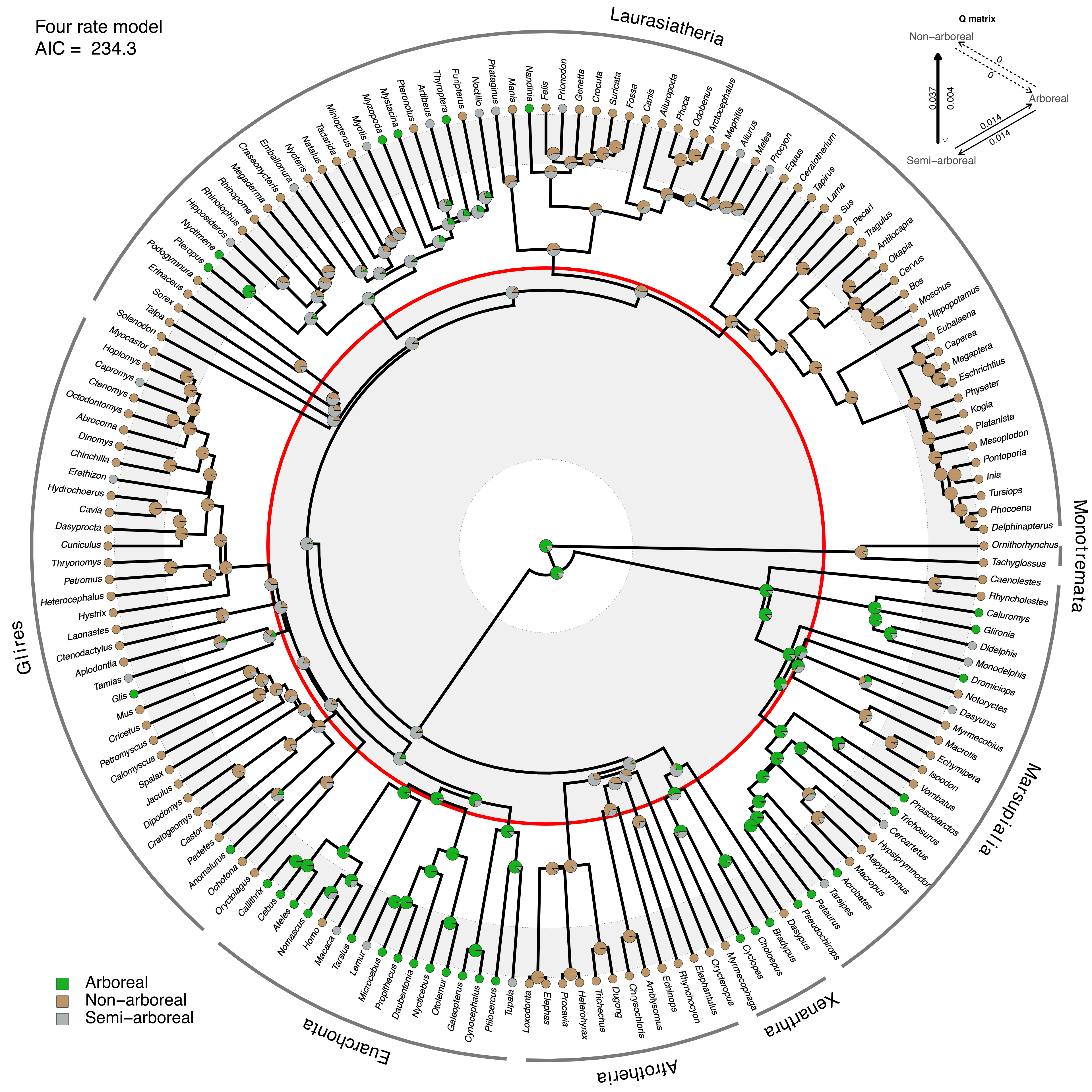

Four rate model
AIC = 234.3
Q matrix
Non−arboreal
Arboreal
Semi−arboreal
0
0
0.037
0.004
0.014
0.014
Laurasiatheria
Monotremata
Marsupialia
Xenarthra
Afrotheria
Euarchonta
Glires
Podogymnura
Pteropus
Nyctimene
Hipposideros
Rhinolophus
Rhinopoma
Megaderma
Craseonycteris
Emballonura
Nycteris
Natalus
Tadarida
Miniopterus
Myotis
Myzopoda
Mystacina
Pteronotus
Artibeus
Thyroptera
Furipterus
Noctilio
Phataginus
Manis
Nandinia
Felis
Prionodon
Genetta
Crocuta
Suricata
Fossa
Canis
Ailuropoda
Phoca
Odobenus
Arctocephalus
Mephitis
Ailurus
Meles
Procyon
Equus
Ceratotherium
Tapirus
Lama
Sus
Pecari
Tragulus
Antilocapra
Okapia
Cervus
Bos
Moschus
Hippopotamus
Eubalaena
Caperea
Megaptera
Eschrichtius
Physeter
Kogia
Platanista
Mesoplodon
Pontoporia
Inia
Tursiops
Phocoena
Delphinapterus
Ornithorhynchus
Tachyglossus
Caenolestes
Rhyncholestes
Caluromys
Glironia
Didelphis
Monodelphis
Dromiciops
Notoryctes
Dasyurus
Myrmecobius
Macrotis
Echymipera
Isoodon
Vombatus
Phascolarctos
Trichosurus
Cercartetus
Hypsiprymnodon
Aepyprymnus
Macropus
Acrobates
Tarsipes
Petaurus
Pseudochirops
Dasypus
Bradypus
Choloepus
Cyclopes
Myrmecophaga
Orycteropus
Elephantulus
Rhynchocyon
Echinops
Amblysomus
Chrysochloris
Dugong
Trichechus
Heterohyrax
Procavia
Elephas
Loxodonta
Tupaia
Ptilocercus
Cynocephalus
Galeopterus
Otolemur
Nycticebus
Daubentonia
Propithecus
Microcebus
Lemur
Tarsius
Macaca
Homo
Nomascus
Ateles
Cebus
Callithrix
Oryctolagus
Ochotona
Anomalurus
Pedetes
Castor
Cratogeomys
Dipodomys
Jaculus
Spalax
Calomyscus
Petromyscus
Cricetus
Mus
Glis
Tamias
Aplodontia
Ctenodactylus
Laonastes
Hystrix
Heterocephalus
Petromus
Thryonomys
Cuniculus
Dasyprocta
Cavia
Hydrochoerus
Erethizon
Chinchilla
Dinomys
Abrocoma
Octodontomys
Ctenomys
Capromys
Hoplomys
Myocastor
Solenodon
Talpa
Sorex
Erinaceus
Arboreal
Non−arboreal
Semi−arboreal

Figure S14: Bayesian ancestral state reconstruction of substrate preference in mammals. Pie charts indicate posterior probabilities of character states from SIMMAP under our four-rate model. Topology is taken from Upham et al. (2019) consensus phylogeny. The red circle indicates the K–Pg boundary. Euarchonta and Marsupialia are recovered as arboreal across this boundary, and all other mammalian clades are assigned as semi-arboreal or marginally non-arboreal across the K–Pg boundary.

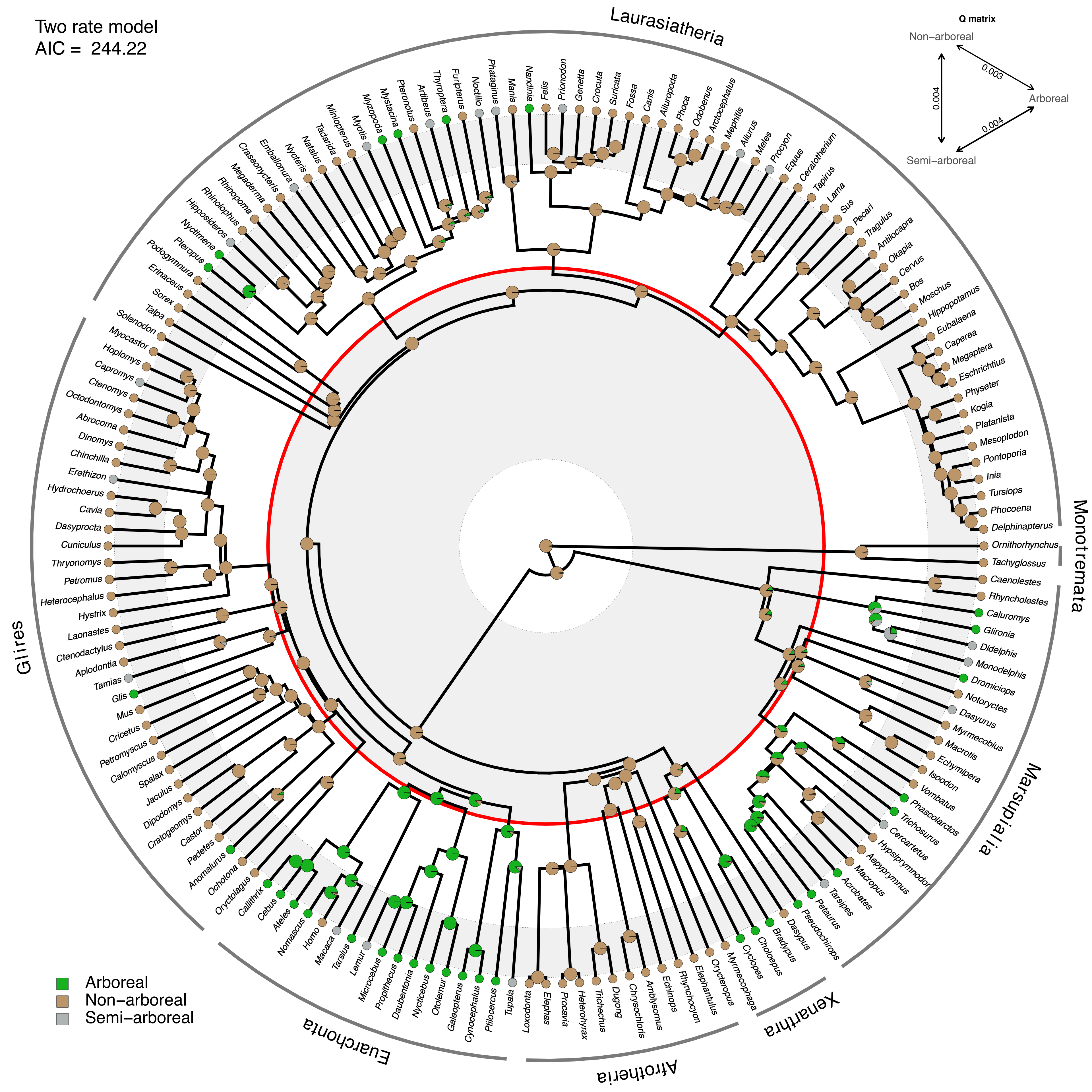

Two rate model
AIC = 244.22
Q matrix
Non–arboreal
Arboreal
Semi–arboreal
0.003
0.004
0.004
Laurasiatheria
Monotremata
Marsupialia
Xenarthra
Afrotheria
Euarchonta
Glires
Podogymnura
Pteropus
Nyctimene
Hipposideros
Rhinolophus
Rhinopoma
Megaderma
Craseonycteris
Emballonura
Nycteris
Natalus
Tadarida
Miniopterus
Myotis
Myzopoda
Mystacina
Pteronotus
Artibeus
Thyroptera
Furipterus
Noctilio
Phataginus
Manis
Nandinia
Felis
Prionodon
Genetta
Crocuta
Suricata
Fossa
Canis
Ailuropoda
Phoca
Odobenus
Arctocephalus
Mephitis
Ailurus
Meles
Procyon
Equus
Ceratotherium
Tapirus
Lama
Sus
Pecari
Tragulus
Antilocapra
Okapia
Cervus
Bos
Moschus
Hippopotamus
Eubalaena
Caperea
Megaptera
Eschrichtius
Physeter
Kogia
Platanista
Mesoplodon
Pontoporia
Inia
Tursiops
Phocoena
Delphinapterus
Ornithorhynchus
Tachyglossus
Caenolestes
Rhyncholestes
Caluromys
Glironia
Didelphis
Monodelphis
Dromiciops
Notoryctes
Dasyurus
Myrmecobius
Macrotis
Echymipera
Isoodon
Vombatus
Phascolarctos
Trichosurus
Cercartetus
Hypsiprymnodon
Aepyprymnus
Macropus
Acrobates
Tarsipes
Petaurus
Pseudochirops
Dasypus
Bradypus
Choloepus
Cyclopes
Myrmecophaga
Orycteropus
Elephantulus
Rhynchocyon
Echinops
Amblysomus
Chrysochloris
Dugong
Trichechus
Heterohyrax
Procavia
Elephas
Loxodonta
Tupaia
Ptilocercus
Cynocephalus
Galeopterus
Otolemur
Nycticebus
Daubentonia
Propithecus
Microcebus
Lemur
Tarsius
Macaca
Homo
Nomascus
Ateles
Cebus
Callithrix
Oryctolagus
Ochotona
Anomalurus
Pedetes
Castor
Cratogeomys
Dipodomys
Jaculus
Spalax
Calomyscus
Petromyscus
Cricetus
Mus
Glis
Tamias
Aplodontia
Ctenodactylus
Laonastes
Hystrix
Heterocephalus
Petromus
Thryonomys
Cuniculus
Dasyprocta
Cavia
Hydrochoerus
Erethizon
Chinchilla
Dinomys
Abrocoma
Octodontomys
Ctenomys
Capromys
Hoplomys
Myocastor
Solenodon
Talpa
Sorex
Erinaceus
Arboreal
Non–arboreal
Semi–arboreal

Figure S15: Bayesian ancestral state reconstruction of substrate preference in mammals. Pie charts indicate posterior probabilities of character states from SIMMAP under our two-rate model. Topology is taken from Upham et al. (2019) consensus phylogeny. The red circle indicates the K–Pg boundary. Euarchonta is recovered as arboreal across this boundary, and all other mammalian clades are assigned as non-arboreal across the K–Pg boundary.

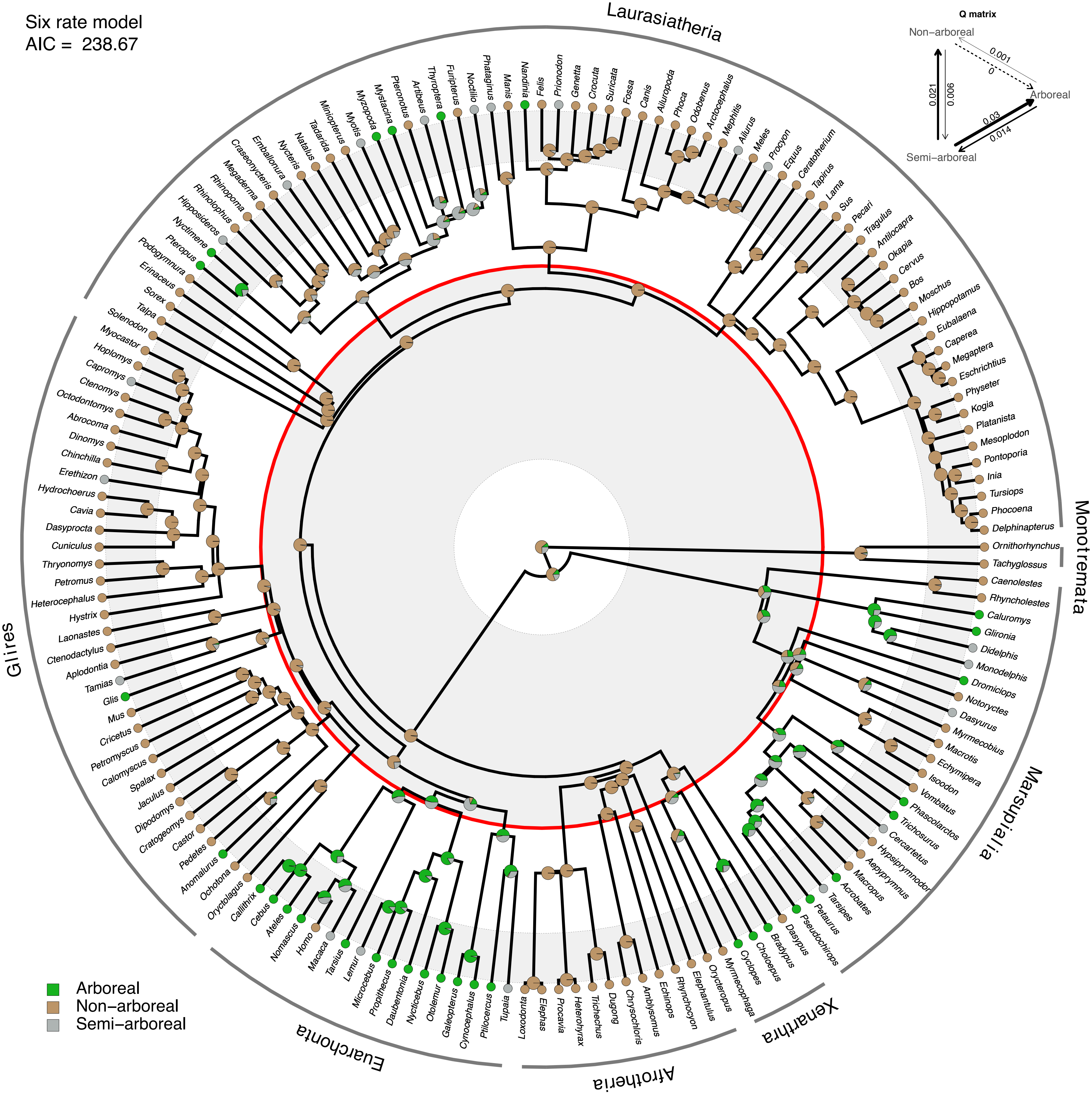

Six rate model
AIC = 238.67
Q matrix
Non−arboreal
Arboreal
Semi−arboreal
0.001
0
0.021
0.006
0.03
0.014
Laurasiatheria
Monotremata
Marsupialia
Xenarthra
Afrotheria
Euarchonta
Glires
Ornithorhynchus
Tachyglossus
Caenolestes
Rhyncholestes
Caluromys
Glironia
Didelphis
Monodelphis
Dromiciops
Notoryctes
Dasyurus
Myrmecobius
Macrotis
Echymipera
Isoodon
Vombatus
Phascolarctos
Trichosurus
Cercartetus
Hypsiprymnodon
Aepyprymnus
Macropus
Acrobates
Tarsipes
Petaurus
Pseudochirops
Dasypus
Bradypus
Choloepus
Cyclopes
Myrmecophaga
Orycteropus
Elephantulus
Rhynchocyon
Echinops
Amblysomus
Chrysochloris
Dugong
Trichechus
Heterohyrax
Procavia
Elephas
Loxodonta
Tupaia
Ptilocercus
Cynocephalus
Galeopterus
Otolemur
Nycticebus
Daubentonia
Propithecus
Microcebus
Lemur
Tarsius
Macaca
Homo
Nomascus
Ateles
Cebus
Callithrix
Oryctolagus
Ochotona
Anomalurus
Pedetes
Castor
Cratogeomys
Dipodomys
Jaculus
Spalax
Calomyscus
Petromyscus
Cricetus
Mus
Glis
Tamias
Aplodontia
Ctenodactylus
Laonastes
Hystrix
Heterocephalus
Petromus
Thryonomys
Cuniculus
Dasyprocta
Cavia
Hydrochoerus
Erethizon
Chinchilla
Dinomys
Abrocoma
Octodontomys
Ctenomys
Capromys
Hoplomys
Myocastor
Solenodon
Talpa
Sorex
Erinaceus
Podogymnura
Pteropus
Nyctimene
Hipposideros
Rhinolophus
Rhinopoma
Megaderma
Craseonycteris
Emballonura
Nycteris
Natalus
Tadarida
Miniopterus
Myotis
Myzopoda
Mystacina
Pteronotus
Artibeus
Thyroptera
Furipterus
Noctilio
Phataginus
Manis
Nandinia
Felis
Prionodon
Genetta
Crocuta
Suricata
Fossa
Canis
Ailuropoda
Phoca
Odobenus
Arctocephalus
Mephitis
Ailurus
Meles
Procyon
Equus
Ceratotherium
Tapirus
Lama
Sus
Pecari
Tragulus
Antilocapra
Okapia
Cervus
Bos
Moschus
Hippopotamus
Eubalaena
Caperea
Megaptera
Eschrichtius
Physeter
Kogia
Platanista
Mesoplodon
Pontoporia
Inia
Tursiops
Phocoena
Delphinapterus
Arboreal
Non−arboreal
Semi−arboreal

Figure S16: Bayesian ancestral state reconstruction of substrate preference in mammals. Pie charts indicate posterior probabilities of character states from SIMMAP under our ARD model. Topology is taken from Upham et al. (2019) consensus phylogeny. The red circle indicates the K–Pg boundary. Euarchonta and Marsupialia are recovered as semi-arboreal across this boundary, and all other mammalian clades are assigned as non-arboreal.

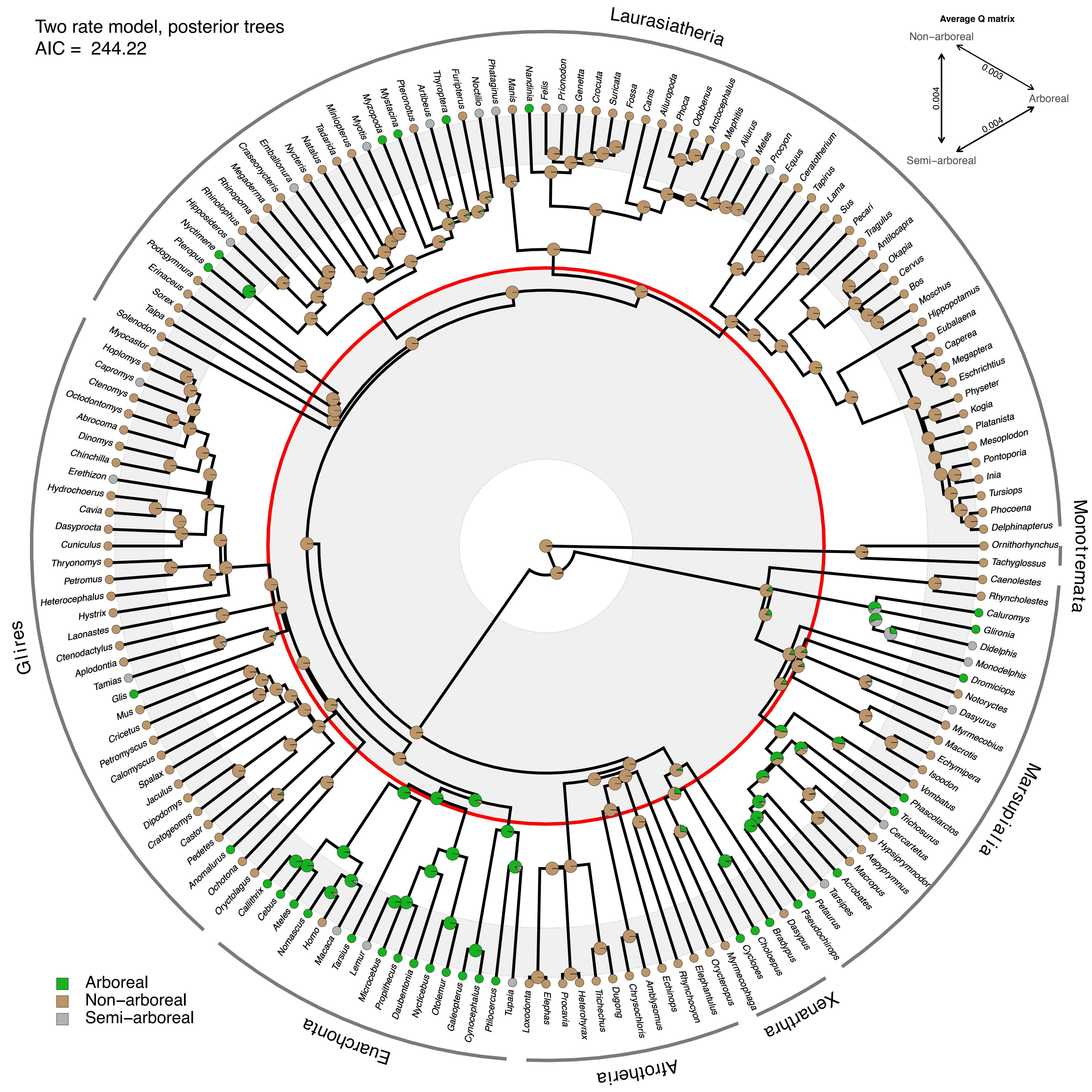

Two rate model, posterior trees
AIC = 244.22
Average Q matrix
Non–arboreal
Arboreal
Semi–arboreal
0.003
0.004
0.004
Laurasiatheria
Monotremata
Marsupialia
Xenarthra
Afrotheria
Euarchonta
Glires
Arboreal
Non–arboreal
Semi–arboreal

Figure S17: Bayesian ancestral state reconstruction of substrate preference in mammals. Pie charts indicate posterior probabilities of character states from SIMMAP under our two-rate model, averaged from 500 simulations across 1,000 sampled posterior trees from Upham et al. (2019). The red circle indicates the K–Pg boundary. Euarchonta is recovered as arboreal across this boundary, and all other mammalian clades are assigned as non-arboreal across it.

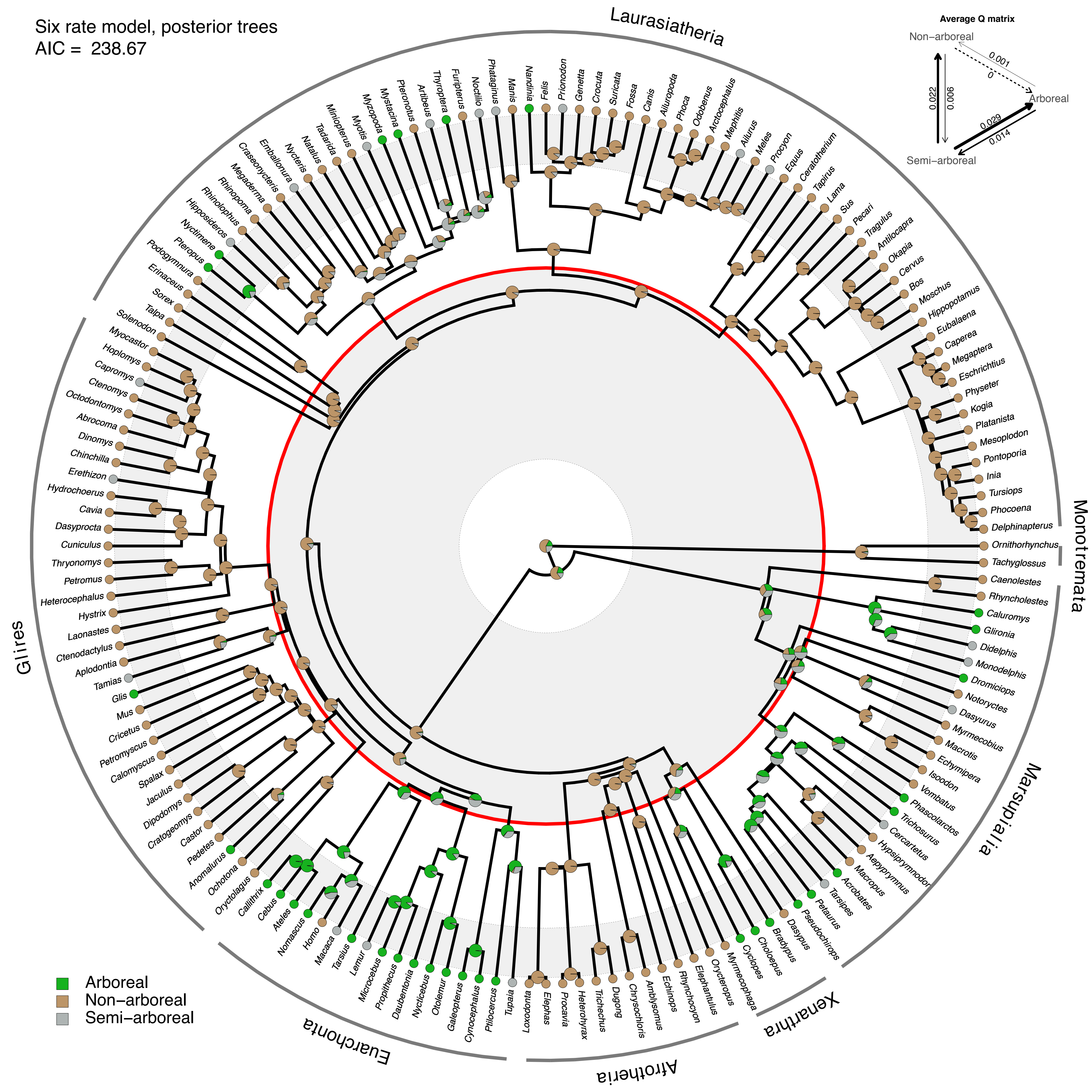

Six rate model, posterior trees
AIC = 238.67
Average Q matrix
Non−arboreal
Arboreal
Semi−arboreal
0.001
0
0.022
0.006
0.029
0.014
Laurasiatheria
Monotremata
Marsupialia
Xenarthra
Afrotheria
Euarchonta
Glires
Arboreal
Non−arboreal
Semi−arboreal
Ornithorhynchus
Tachyglossus
Caenolestes
Rhyncholestes
Caluromys
Glironia
Didelphis
Monodelphis
Dromiciops
Notoryctes
Dasyurus
Myrmecobius
Macrotis
Echymipera
Isoodon
Vombatus
Phascolarctos
Trichosurus
Cercartetus
Hypsiprymnodon
Aepyprymnus
Macropus
Acrobates
Tarsipes
Petaurus
Pseudochirops
Dasypus
Bradypus
Choloepus
Cyclopes
Myrmecophaga
Orycteropus
Elephantulus
Rhynchocyon
Echinops
Amblysomus
Chrysochloris
Dugong
Trichechus
Heterohyrax
Procavia
Elephas
Loxodonta
Tupaia
Ptilocercus
Cynocephalus
Galeopterus
Otolemur
Nycticebus
Daubentonia
Propithecus
Microcebus
Lemur
Tarsius
Macaca
Homo
Nomascus
Ateles
Cebus
Callithrix
Oryctolagus
Ochotona
Anomalurus
Pedetes
Castor
Cratogeomys
Dipodomys
Jaculus
Spalax
Calomyscus
Petromyscus
Cricetus
Mus
Glis
Tamias
Aplodontia
Ctenodactylus
Laonastes
Hystrix
Heterocephalus
Petromus
Thryonomys
Cuniculus
Dasyprocta
Cavia
Hydrochoerus
Erethizon
Chinchilla
Dinomys
Abrocoma
Octodontomys
Ctenomys
Capromys
Hoplomys
Myocastor
Solenodon
Talpa
Sorex
Erinaceus
Podogymnura
Pteropus
Nyctimene
Hipposideros
Rhinolophus
Rhinopoma
Megaderma
Craseonycteris
Emballonura
Nycteris
Natalus
Tadarida
Miniopterus
Myotis
Myzopoda
Mystacina
Pteronotus
Artibeus
Thyroptera
Furipterus
Noctilio
Phataginus
Manis
Nandinia
Felis
Prionodon
Genetta
Crocuta
Suricata
Fossa
Canis
Ailuropoda
Phoca
Odobenus
Arctocephalus
Mephitis
Ailurus
Meles
Procyon
Equus
Ceratotherium
Tapirus
Lama
Sus
Pecari
Tragulus
Antilocapra
Okapia
Cervus
Bos
Moschus
Hippopotamus
Eubalaena
Caperea
Megaptera
Eschrichtius
Physeter
Kogia
Platanista
Mesoplodon
Pontoporia
Inia
Tursiops
Phocoena
Delphinapterus

Figure S18: Bayesian ancestral state reconstruction of substrate preference in mammals. Pie charts indicate posterior probabilities of character states from SIMMAP under our ARD model, averaged from 500 simulations across 1,000 sampled posterior trees from Upham et al. (2019). The red circle indicates the K–Pg boundary. Euarchonta is recovered as arboreal across this boundary, and all other mammalian clades are assigned as non-arboreal across it.
